# Neuronal modulation in the mouse superior colliculus during covert visual selective attention

**DOI:** 10.1038/s41598-022-06410-5

**Published:** 2022-02-15

**Authors:** Lupeng Wang, James P. Herman, Richard J. Krauzlis

**Affiliations:** 1 Laboratory of Sensorimotor Research, National Eye Institute, Bethesda, MD 20892 USA; 2 Department of Ophthalmology, University of Pittsburgh, Pittsburgh, PA 15213 USA

**Keywords:** Neuroscience, Cognitive neuroscience, Sensorimotor processing, Sensory processing, Visual system

## Abstract

Covert visual attention is accomplished by a cascade of mechanisms distributed across multiple brain regions. Visual cortex is associated with enhanced representations of relevant stimulus features, whereas the contributions of subcortical circuits are less well understood but have been associated with selection of relevant spatial locations and suppression of distracting stimuli. As a step toward understanding these subcortical circuits, here we identified how neuronal activity in the intermediate layers of the superior colliculus (SC) of head-fixed mice is modulated during covert visual attention. We found that spatial cues modulated both firing rate and spike-count correlations. Crucially, the cue-related modulation in firing rate was due to enhancement of activity at the cued spatial location rather than suppression at the uncued location, indicating that SC neurons in our task were modulated by an excitatory or disinhibitory circuit mechanism focused on the relevant location, rather than broad inhibition of irrelevant locations. This modulation improved the neuronal discriminability of visual-change-evoked activity, but only when assessed for neuronal activity between the contralateral and ipsilateral SC. Together, our findings indicate that neurons in the mouse SC can contribute to covert visual selective attention by biasing processing in favor of locations expected to contain task-relevant information.

## Introduction

Visual selective attention is achieved through distributed neuronal circuits that include cortical and subcortical areas. Our understanding of these circuits mainly comes from neurophysiological studies in non-human primates that manipulate attention using informative cues. For instance, cortical neurons, including those in the visual cortex^1^, frontal^2^ and parietal cortices^3^, display cue-related modulation marked by increases in firing rate^4^ and changes in the correlated variability^5^, both of which can affect the decoding of visual information. In subcortical areas, neurons in primate superior colliculus^6^, thalamus^7,8^ and caudate nucleus of the basal ganglia^9^ also display cue-related modulation during attention tasks. In contrast to cortical attention mechanisms that appear to regulate the quality of local visual processing, subcortical circuits have been implicated in the spatial weighting of visual signals^10^ and the suppression of distractors^11,12^.

The mouse has emerged as an important model for studying the detailed neuronal mechanisms for visual functions, because of unparalleled access to genetic tools. Complementing these tools, recent behavioral studies have demonstrated the feasibility of studying visual selective attention in mice^13–15^. Notably, we reported that mice display perceptual benefits from informative spatial cues, a well-known attentional effect^13^. However, how neuronal activity in the mouse brain is modulated during visual attention tasks is largely unknown.

The midbrain superior colliculus (SC) is a crucial subcortical structure for the control of visual selective attention^10,16^, and provided the first demonstrations of attention-related changes in neuronal activity^17^. Whereas studies of mouse SC visual functions have largely focused on innate orienting behaviors, including predator avoidance or prey approach^18^, we recently found that inhibiting visually evoked SC activity in mice impairs their voluntary visual perceptual choices, and that the perceptual impairment was larger when a competing visual stimulus was present^19^, consistent with a role of the mouse SC in visual selection^20^. However, the involvement of the mouse SC in attention itself has not yet been explicitly tested—the mouse SC could be involved in prioritizing task-relevant spatial locations, or suppressing distractors, or possibly only involved in earlier stages of visual processing that precede these aspects of attention.

Here we investigated the neuronal correlates of visual selective attention in the intermediate layers of the mouse SC by recording the spiking activity of neurons during a visual orientation change-detection task and using spatial cues to manipulate the allocation of selective attention. We used a task design identical to that used recently to demonstrate that mice can exhibit covert visual selective attention^13^, adapted from classic covert attention paradigms in humans. First, to isolate covert attention, we used head-fixed mice and an apparatus that required mice to attend to visual stimuli without overtly orienting toward them. Second, to manipulate the allocation of spatial attention, we varied whether mice were provided spatial cues across blocks of trials—while keeping the visual stimulation identical. Using this approach, we found that visual activity in the mouse SC displayed cue-related modulation, including changes in spike rate and interneuronal spike-count correlations. By comparing activity across attention task conditions, we determined that cue-related modulation in our experiment was the result of enhancement at the cued spatial location rather than suppression at the uncued location. Together, our results demonstrate how SC neurons can contribute to attention in the mouse by biasing signal processing in favor of spatial locations expected to contain behaviorally relevant events.

## Results

**Figure 1:**
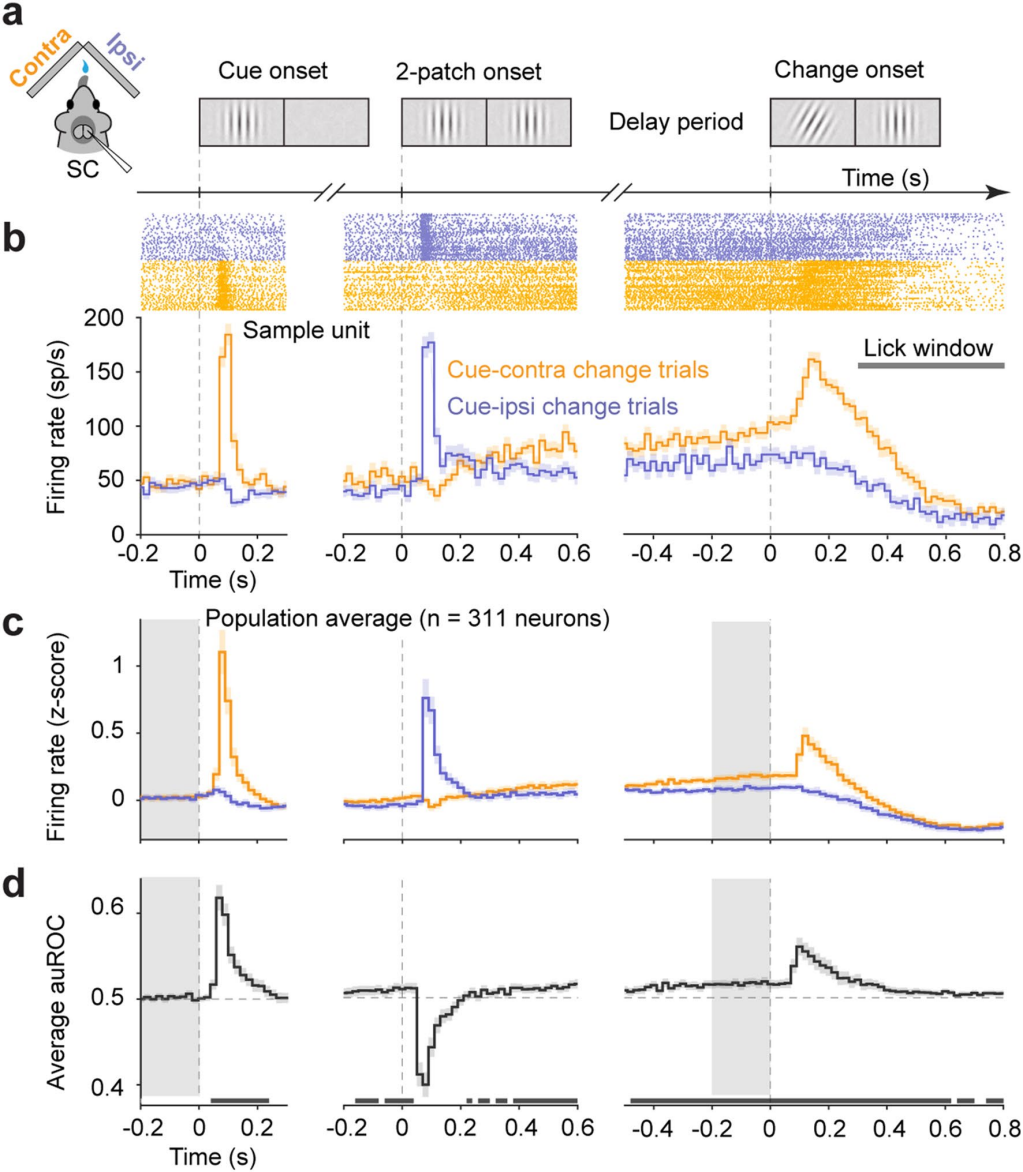
Spatial cue related modulation of mouse SC neuronal activity in a visual change detection task. (**a**) Schematics of unilateral recording in the mouse SC during the “contra/ipsi cue” orientation change detection task, and illustration of visual stimuli on two visual displays in sequences of task epochs: cue, 2 patch and change. Delay period refers to the entire 2-patch epoch. (**b**) Firing rates of a sample SC unit aligned on three task epochs, shown as spike raster (upper) of cue-contra change trials (orange) and cue-ipsi change trials (blue) and Peristimulus time histograms (PSTHs, lower) in 20-ms bins, only trials with hit were plotted. Gray horizontal bar indicates lick window from 300 to 800 ms after orientation change. Shades are error bars, representing 95% confidence interval [CI] of the mean. (**c**) Normalized population PSTHs aligned on the onset of three epochs. Plotting conventions as in (**b**). Gray area in the left panel indicates the 200 ms period precedes the cue-patch onset used to calculate baseline activity; gray area in the right panel indicates the final 200 ms of the 2-patch epoch (defined as the specific analysis “delay period”). (**d**) Time course of population average area under ROC (auROC) comparing spike counts between cue-contra change and cue-ipsi change trials aligned on three task epochs in 20-ms bins. The row of gray boxes below mark bins in which population auROC are significantly > 0.5 chance level (*p* < 0.05), as measured in two-tailed Wilcoxon signed-rank tests on population auROC values. The gray area in the left and right panels has the same convention as in (**c**).

To investigate cue-related modulation of mouse SC neuronal activity, we recorded the activity of SC neurons in two variants of a spatial cueing task. The main task, which we term “contra/ipsi cue”, was similar to one used previously^13,21^. In brief, head-fixed mice viewed stimuli on a pair of lateralized displays while running on a wheel. The animals’ locomotion controlled each trial’s progression through several epochs defined by visual stimulus events (Fig. 1a), and determined the drift rate of Gabor stimuli. Presentation of a single lateralized Gabor patch served as a spatial cue, indicating the potential location of an upcoming orientation change, and defined the start of the “cue epoch”. The appearance of a second Gabor patch in the opposite visual hemifield marked the start of the “2-patch epoch”, throughout which both Gabor patches remained present. This epoch was especially relevant for our study because the visual stimulation was identical, but the spatial cueing differed across conditions; this epoch is often called the “delay period” in the attention literature^22^. In trials with an orientation change (50% of trials), the start of the “change epoch” was marked by a tilt in orientation of the cue patch. Mice were required to lick a center spout within a 500 ms response window to indicate detection of the orientation change and receive a liquid reward. Each session was organized into alternating sub-blocks of 40 left-cue and 40 right-cue trials. We used this task to characterize the spatial specificity and time course of cue-related modulation in mouse SC neurons (n = 94 sessions). In a subset of sessions (n = 25) we also recorded SC neuronal activity in a variant of the main task which included sub-blocks of 80 no-cue trials interleaved with left-cue and right-cue sub-blocks; accordingly, we refer to this variant as the “cue/no-cue” task. Only trials with correct responses were included for analyses of neuronal activity.

Extracellular activity was recorded from neurons in the intermediate and deep SC layers, located at least 400 µm below the dorsal surface of SC (Fig. S1), using moveable chronic 16-channel microwire bundles. We analyzed the activity of 311/415 SC neurons with clear visual spatial receptive fields that overlapped the contralateral visual stimulus location (Fig. S2).

### SC neuronal responses to visual events and modulation by spatial cue location

In addition to phasic visual onset responses, many SC neurons displayed cue-related modulation during the “contra/ipsi cue” attention task but only when the visual stimulus was relevant (Fig. 1). Prior to cue onset, many neurons displayed tonic activity (Fig. 1b,c left panels). This pre-cue baseline activity was not spatially modulated; the pre-cue spike rate was statistically indistinguishable between the two cue locations (population mean spike count within a 200 ms interval before cue onset: contralateral: 2.40 ± 0.34, mean ± 95% confidence interval [CI]; ipsilateral: 2.36 ± 0.32; *p* = 0.84, paired-sample Wilcoxon signed-rank test).

The onset of the spatial cue (“cue onset”) evoked a phasic response in most neurons (81%, 251/311, significant units, see “Methods”) when it was presented contralaterally (contra-cue), and brief inhibition when presented ipsilaterally (ipsi-cue). The onset of the second Gabor patch (“2-patch onset”) had a similar effect, causing a phasic increase in activity when presented contralaterally in ipsi-cue trials and a decrease when presented ipsilaterally in contra-cue trials (Fig. 1c).

Following stimulus onset transients, neuronal activity in contra-cue trials gradually increased over time. On average, contra-cue activity exceeded activity in ipsi-cue trials ~ 250 ms after the start of the 2-patch epoch and remaining elevated throughout the remainder of that epoch. We identified this relative elevation of neuronal activity in contra-cue trials compared to ipsi-cue trials during the delay period as cue-related modulation for two reasons. First, visual stimuli presented during this interval were identical across the two cue conditions, so the relative elevation of cue-contra activity compared to cue-ipsi depended on the location of the spatial cue presented in the preceding epoch and not on lateralized differences in visual stimulation. Second, this modulation was not a result of lingering visual responses caused by preceding stimulus onset events, because the most recent visual event in contra-cue trials was the appearance of an ipsilateral patch that slightly decreased activity (Fig. 1b,c, middle panel). Instead, the modulation emerged gradually over time during the delay period, under identical visual conditions, on a time scale that anticipated the possible visual change event.

After the near-threshold change in orientation, which remained fixed throughout the rest of the trial, many SC neurons exhibited robust transient increases in activity for contralateral changes (35%, 108 / 311, significant units, see “Methods”) before decreasing activity towards the end of a trial and modest slower activity reductions for ipsilateral changes (Fig. 1b,c, right panels). Note that these change-related increases and decreases in SC activity were superimposed on distinct pre-change levels of neuronal activity due to cue-related modulation during the preceding 2-patch epoch.

To quantify cue-related modulation in single neurons, we used the receiver operating characteristic (ROC) approach from signal detection theory^23,24^. We computed the area under the ROC curve (auROC) comparing spike rates in ipsi-cue trials (“signal absent”) to spike rates in contra-cue trials (“signal present”) in consecutive non-overlapping 20-ms bins (Fig. 1d). Consistent with our comparison of population mean activity above, average auROC across our population of SC neurons indicated that before cue onset, spike rates of SC neurons did not differentiate between contra-cue and ipsi-cue trial types (Fig. 1d left panel). Rather, after a transient suppression of activity caused by the appearance of the ipsilateral patch, spike rates on contra-cue trials became higher than ipsi-cue trials approximately 220 ms after the onset of the 2-patch epoch and remained significantly elevated throughout the rest of the epoch (Fig. 1d middle and right panels). These results demonstrate that, even though spatial cue information was available throughout the block of trials, cue-related modulation of SC neurons emerged only after the visual stimuli were behaviorally relevant.

**Figure 2:**
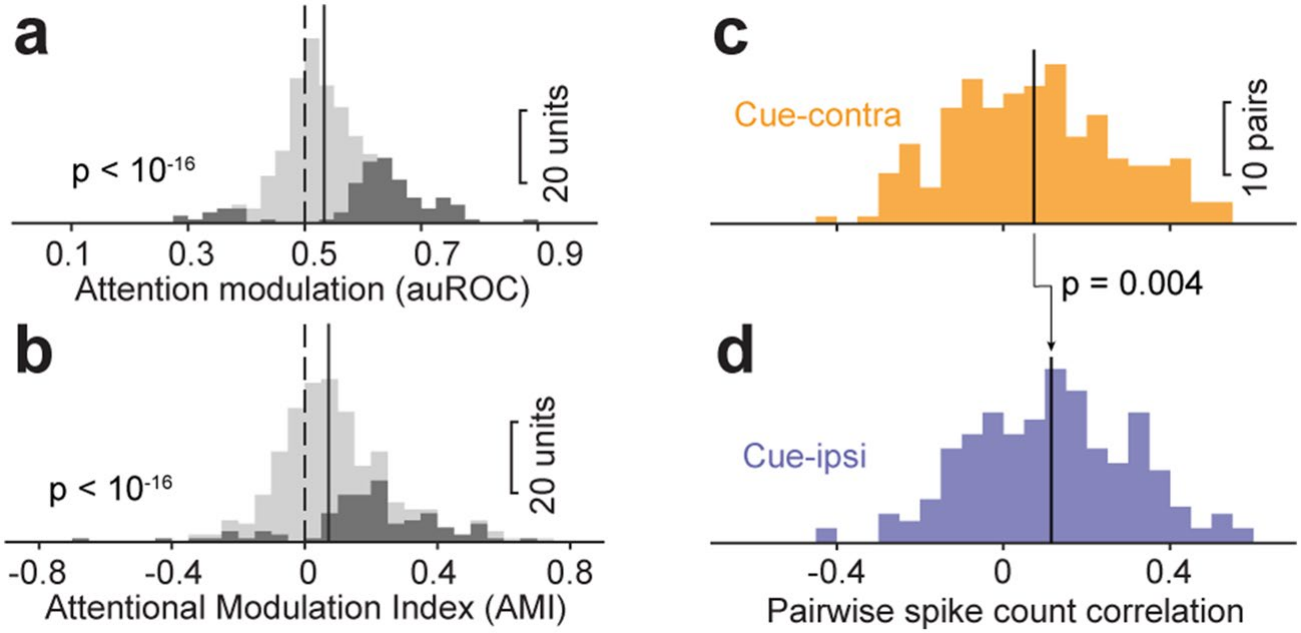
Population summary of cue-related modulation on mouse SC firing rate and interneuronal spike-count correlations. (**a**) Distribution of delay period activity auROC comparing cue-contra and cue-ipsi trials; bin width of histograms is 0.05. Dark bars count units with auROC value significant different from chance level (bootstrapped 95% CI ⊄ 0.5). Dashed line indicates chance value of 0.5, solid line indicates population median (0.53). (**b**) Distribution of delay-period AMIs between contralaterally and ipsilaterally cued trials, using the same conventions as in (**a**). Dark bars count units with significant AMI (*p* < 0.05, rank sum test of spike count between cue-ipsi and cue-contra trials). Dashed line indicates chance value of 0, solid line indicates population median (0.07). (**c**) Distribution of delay period interneuronal spike-count correlations during “cue-contra” trials of simultaneously recorded SC neuronal pairs. Solid line: population median. (**d**) Distribution of delay period spike-count correlations during “cue-ipsi” trials. Solid line: population median. P value is from paired-sample Wilcoxon signed-rank test comparing population medians between “cue-contra” and “cue-ipsi” trials.

To quantify cue-related modulation during the time when stimuli were relevant for guiding behavior, we defined a specific “delay period” as the final 200 ms of the 2-patch epoch immediately preceding the orientation change. Crucially, visual stimuli were identical across contra-cue and ipsi-cue trials during this delay period. Across our sample of SC units, cue-related modulation during the delay period measured by auROC was significantly larger than chance (*p* < 10^–16^, one-tailed Wilcoxon signed-rank test, Fig. 2a), indicating that our population of SC neurons had higher spike rates in contra-cue trials than ipsi-cue trials during this interval (28%, 87/311 of units show auROC values significantly > 0.5 chance level, bootstrapped 95% CI > 0.5). Similar results were obtained using attentional modulation indices, the other widely used measurement of attention related modulation of neuronal activity (Fig. 2b). These results demonstrate that mouse SC neurons exhibit classic cue-related modulation in firing rates.

Spatial cueing can also influence the information available in neuronal populations through effects on the structure of correlated variability amongst neurons^5,25^. To test for this in our mouse SC neurons, we computed spike count (“noise”) correlations during the delay period of cue-contra trials and cue-ipsi trials in simultaneously recorded neuronal pairs (n = 203 pairs). As shown in Fig. 2c,d, the distribution of spike-count correlations across pairs of mouse SC neurons was broad (standard deviation for cue-contra = 0.200; cue-ipsi = 0.196) and on average positive (mean for cue-contra = 0.074; cue-ipsi = 0.115). Notably, spike-count correlations in cue-contra trials (median = 0.073) were significantly smaller than those in cue-ipsi trials (median = 0.115), indicating that the degree of correlated variability was reduced for SC neurons representing the cued location as mice awaited a potential visual event.

Because several other factors might also contribute to modulation of mouse SC neuronal activity during our attention task, including behavioral states and locomotion^26,27^, we assessed the influence of these factors, as well as the spatial cue condition, on spike rates of individual SC neurons using linear regression analysis (Fig. S3). This analysis revealed that some mouse SC neurons were indeed significantly modulated by running speed (29%, 90/311, significant units, see “Methods”) or pupil size (27%, 83/311). However, the influence of these factors on spike rate was manyfold smaller than that of spatial cue condition, which was the single largest contributor (p < 10^–9^ for both Tukey–Kramer post-hoc comparison tests following one-way ANOVA on linear regression coefficients) to variation in spike rate during the delay period. These results reinforce our conclusion that spatial cue information was specifically important in modulating SC neurons during the visual attention task.

### Cue-related modulation resulted mostly from enhanced activity at the location of expected visual events

The relative difference in spike rate between contra-cue and ipsi-cue trials during the delay period could be due to enhancement of processing at the spatial location expected to contain behaviorally relevant information, or suppression of activity at locations not expected to contain such information, or a combination of both effects. In the following, we first quantify the cue-related enhancement and suppression during the delay period when stimulus onsets and behavioral responses were absent, allowing the clearest view of cue-related modulatory effects alone. We will then also consider enhancement and suppression in the change epoch.

**Figure 3:**
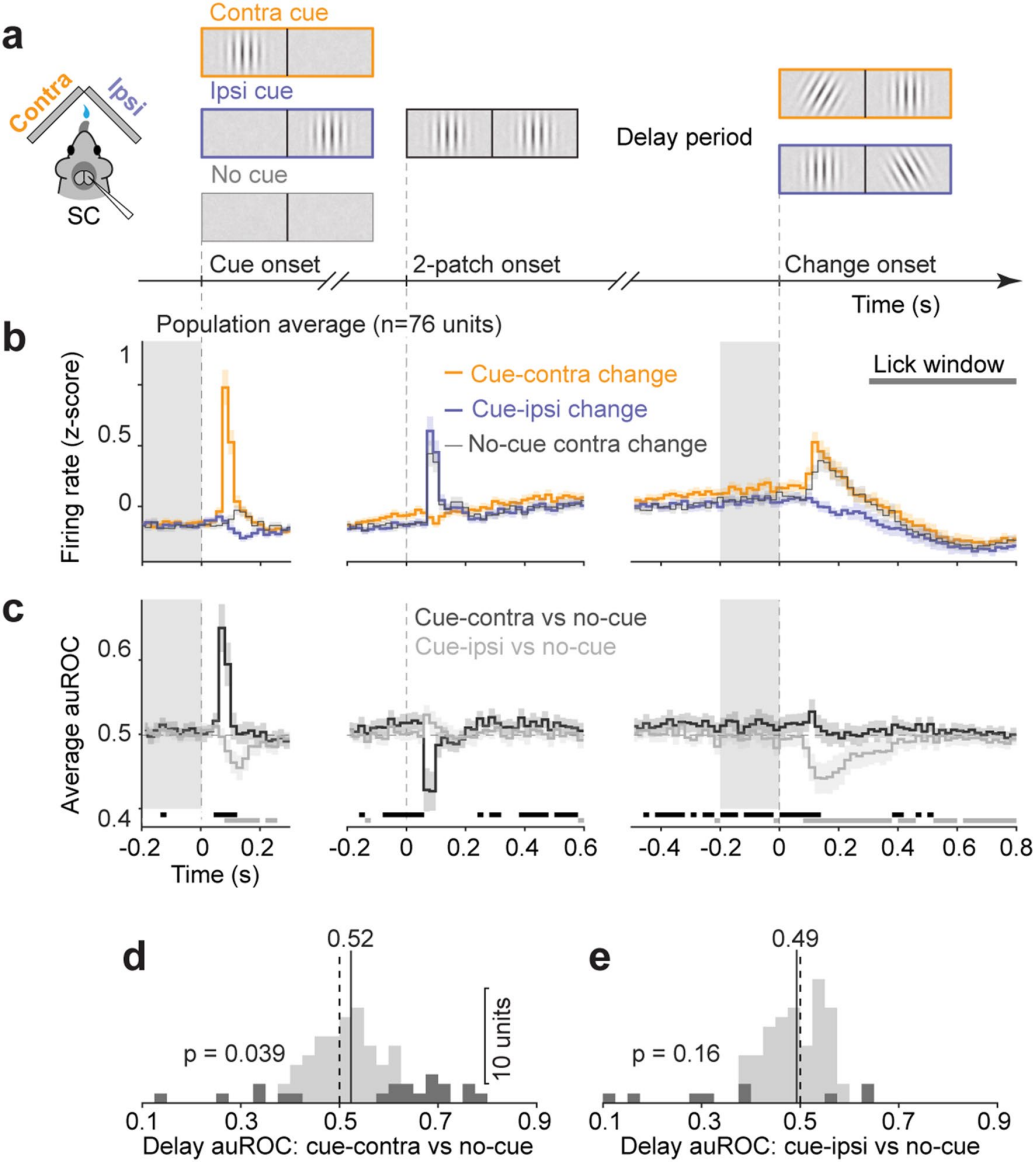
Cue-related modulation resulted from enhanced activity at contralaterally cued spatial locations. (**a**) Schematic of epochs in “cue/no-cue” task, where no-cue trials had cue epoch replaced with a noise epoch and orientation change could occur either left or right with equal frequency. No-cue sub-blocks were interleaved with cued sub-blocks. (**b**) Normalized population PSTHs of SC neurons for cue-contra change (orange), cue-ipsi change (blue) and no-cue contra change trials (gray), aligned on the onset of task epochs. Error bars: 95% CI of mean. Gray area indicates the delay period used for analysis shown in (**d**,**e**). Gray horizontal bar indicates the 500-ms lick window. (**c**) Time course of population average auROCs comparing “cue-contra” spike counts to “no-cue” change trials (dark), and comparing “cue-ipsi” to “no-cue” change trials (gray), aligned on three task epochs in 20 ms bins. Only “no-cue” trials with contralateral orientation change were illustrated. The row of dark gray boxes below mark bins in which population auROCs for cue-contra vs no-cue are significantly > 0.5 chance level (*p* < 0.05), as measured in two-tailed Wilcoxon signed-rank test on population auROC values; row of light gray boxes below mark bins in which population auROC for cue-ipsi vs no-cue are significantly < 0.5 chance level (*p* < 0.05), as measured in two-tailed Wilcoxon signed-rank test on population auROC values. (**d**) Distribution of delay period auROCs comparing “cue-contra” and “no-cue” change trials. Dark bars count units with auROC values significantly different from chance level (bootstrapped 95% CI ⊄ 0.5). Dashed line indicates chance value of 0.5, solid line with value indicates population median. (**e**) Presentation as in (**d**), but for auROCs comparing “cue-ipsi” to “no-cue” trials.

To address the roles of cue-related enhancement and suppression, we used a “cue/no-cue” variant of our attention task^13^ that allowed us to compare SC neuronal activity evoked by cued and uncued stimuli to activity evoked by identical visual stimuli but presented in a context with no spatial cueing. For no-cue trials in these experiments, the cue-epoch was replaced with the same pink noise present prior to cue onset in the “cue-contra/cue-ipsi” task and was then followed by our standard 2-patch epoch (Fig. 3a). Orientation changes in these no-cue trials occurred in pseudorandom order on the left or right with equal frequency.

We first verified that providing the spatial cue improved behavioral performance. Perceptual sensitivity, measured using the behavioral metric d’ from signal detection theory, was significantly higher on with-cue trials than no-cue trials (with-cue: 1.64 ± 0.11, mean ± SEM, n = 25 sessions; no-cue: 1.36 ± 0.11, p < 0.001, Paired-sample Wilcoxon signed-rank test); decision criterion was significantly lower on with-cue trials than no-cue trials (with cue: − 0.36 ± 0.11; no-cue: − 0.15 ± 0.13, p = 0.004), consistent with our previous results^13^. Similarly, reaction time and response accuracy also significantly improved on with-cue trials (Figure S4).

Having established the behavioral benefits of providing a spatial cue, we next tested how spatial cues altered SC activity. As expected, SC activity in with-cue trials during the “cue/no-cue” task (Fig. 3b) recapitulated the cue-related modulation found during the “contra/ipsi cue” task (Fig. 1c), with greater delay period activity in cue-contra compared to cue-ipsi trials. Also, we again found no significant variation in pre-cue baseline activity across cueing conditions (population mean spike count within a 200-ms pre-cue interval: cue-contra: 2.79 ± 0.71, mean ± 95% CI; cue-ipsi: 2.70 ± 0.69; no-cue: 2.61 ± 0.67; p = 0.98, non-parametric Kruskal–Wallis one-way ANOVA, Fig. 3c left panel). Comparison of activity in no-cue trials to that in both cue-contra and cue-ipsi trials then revealed how these cueing effects arose – namely by enhancement at the cued location, with little or no suppression at the uncued location. As shown by the superimposed traces in Fig. 3b, activity in no-cue trials (thin gray line) was not only lower than the activity in contra-cue trials, but was also nearly identical to activity in ipsi-cue trials (thick blue line). The only exceptions to this pattern were found during transients evoked after the onset of the cue and the contralateral orientation change, which were expected given the difference in visual stimulus conditions at those points in the trial.

To document the time course of cue-related effects, we again computed spike rate auROC values in consecutive 20-ms bins (Fig. 3c), separately comparing contra-cue and ipsi-cue (“signal present”) to the no-cue condition (“signal absent”). Before the cue epoch, neither comparison showed average auROC values significantly different from chance in any bin (Fig. 3c left panel). Average auROC values for contra-cue versus no-cue became significantly greater than chance approximately 240 ms after the onset of the 2-patch epoch and remained significantly elevated for the rest of the delay period, indicating a sustained enhancement of activity at the cued location. In contrast, auROC values for ipsi-cue versus no-cue trials were not different from chance from 120 ms through the remainder of the delay period (Fig. 3c middle and right panels), demonstrating that cueing did not produce suppression at the uncued location.

We additionally quantified cueing effects using multiple measures specifically during the “delay period” (final 200 ms of the 2-patch epoch). The distribution of auROC values was significantly greater than chance when comparing cue-contra to no-cue (*p* = 0.039, two-tailed Wilcoxon signed-rank test, Fig. 3d), but not when comparing cue-ipsi to no-cue (*p* = 0.16, Fig. 3e). We found the same pattern of results when we quantified population cueing effects with an attention modulation index (AMI; cue-contra vs no-cue: 0.058 ± 0.041, *p* = 0.011, two-tailed Wilcoxon signed-rank test; cue-ipsi vs no-cue: − 0.018 ± 0.043, *p* = 0.17), and also when we quantified cueing effects directly from spike counts (population mean spike count: cue-contra: 4.22 ± 0.16; cue-ipsi: 3.86 ± 0.15; no-cue: 4.05 ± 0.13, *p* < 10^–15^, cueing condition effect, two-way ANOVA for trial-by-trial spike counts, using cueing conditions and neuron identity as factors), with contra-cue spike counts the highest (*p* < 10^–8^, compared to both cue-ipsi and no-cue, Tukey–Kramer post-hoc comparison) and no difference between cue-ipsi and no-cue spike counts (*p* = 0.10).

Together, these results demonstrate that the cue-related modulation we observed for mouse SC neurons in our attention task was largely due to the enhancement of processing at the cued spatial location rather than suppression at the uncued location.

### Effects of spatial cues on SC neuronal discriminability of visual events

Having established that spatial cues enhanced delay period neuronal activity specifically at the cued location, we next examined how cueing influenced neuronal activity evoked by the behaviorally relevant orientation-change event. We recently found that unilateral suppression of SC neuronal activity in a short time interval immediately after the orientation change causes major deficits in the ability of mice to correctly detect these near-threshold visual events^19^. Given that SC neuronal activity appears to be crucial for this detection task, we sought to identify how the effects of spatial cueing on SC neurons might contribute to the observed improvements in task performance during the “cue/no-cue” experiment.

**Figure 4:**
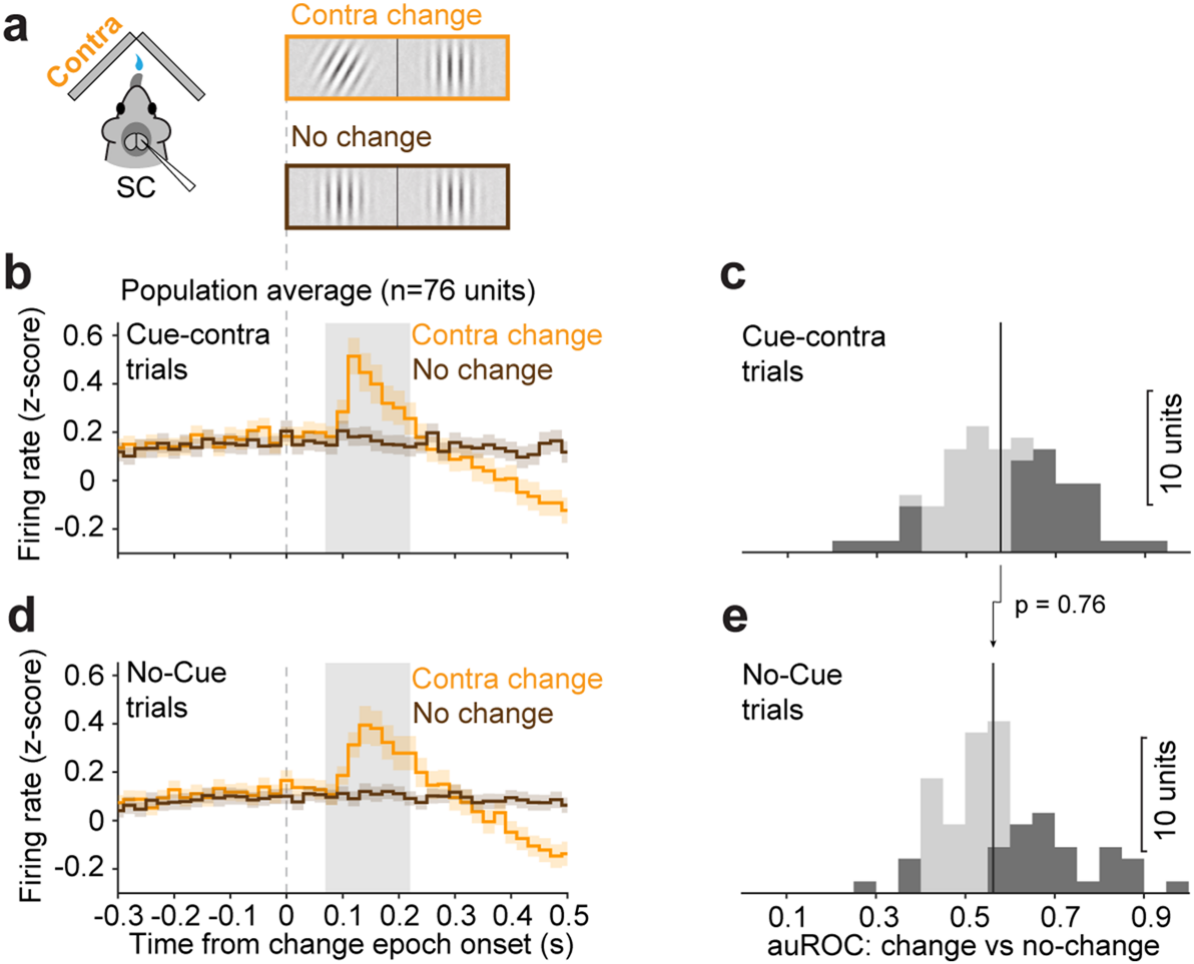
Spatial cueing did not alter the ability of SC neurons to detect contralateral changes. (**a**) Schematic of apparatus and events in “cue/no-cue” task. (**b**) Average normalized population PSTHs of SC neurons during “cue-contra” change trials (orange) and “cue-contra” no-change trials (brown). Traces are aligned on the change onset. Gray area: 150 ms change interval (60–210 ms after change onset) used to compute auROC comparing contralateral change and no-change trials. (**c**) Distribution of change vs no-change auROCs in contralaterally cued trials. Dark bars are units with auROCs significantly different from chance level. Dashed line indicates chance value of 0.5, solid line indicates population median (0.58). (**d**) Presentation as in (**b**) but in no-cue trials, comparing “no-cue contra” change (orange) to “no-cue” no-change (brown) trials. (**e**) Presentation as in (**c**), but in no-cue trials; (median = 0.56). The p value indicates comparison of population median between (**c**) and (**e**), paired-sample Wilcoxon signed-rank test.

One possibility is that spatial cueing improves the ability of mouse SC neurons to discriminate between change and no-change events in their receptive fields (Fig. 4a), as has been observed in primate visual cortex^28^. However, we found no evidence that spatial cueing affected neuronal discriminability for contralateral events (Fig. 4). We computed auROC values for a “change” window (150 ms interval beginning 60 ms after the change, or a matched interval in trials with no change; see “Methods”) by comparing spike counts in contra-change versus no-change trials, separately for contra-cue (Fig. 4b,c) and no-cue trials (Fig. 4d,e). There was no significant difference between auROC values from the contra-cue condition compared to no-cue (*p* = 0.76, paired-sample Wilcoxon signed-rank test), demonstrating that cueing did not improve SC neurons’ discrimination of orientation changes versus non-changes occurring in their receptive fields. Indeed, we found both that change-window spike counts in cue-contra contra-change trials were significantly higher than those in no-cue contra-change trials (medians: 4.02 vs. 3.75, *p* = 0.04, paired-sample Wilcoxon signed-rank test), and that spike counts in cue-contra no-change trials were significantly higher than those in no-cue no-change trials (medians: 3.16 vs. 2.94, *p* = 0.02). These results indicate that the existing elevation of activity during the delay period, due to the contralateral cue, persisted through the change epoch regardless of the presence or absence of a contralateral change event, consistent with the spatial cueing producing an offset in SC activity for the cued location.

**Figure 5:**
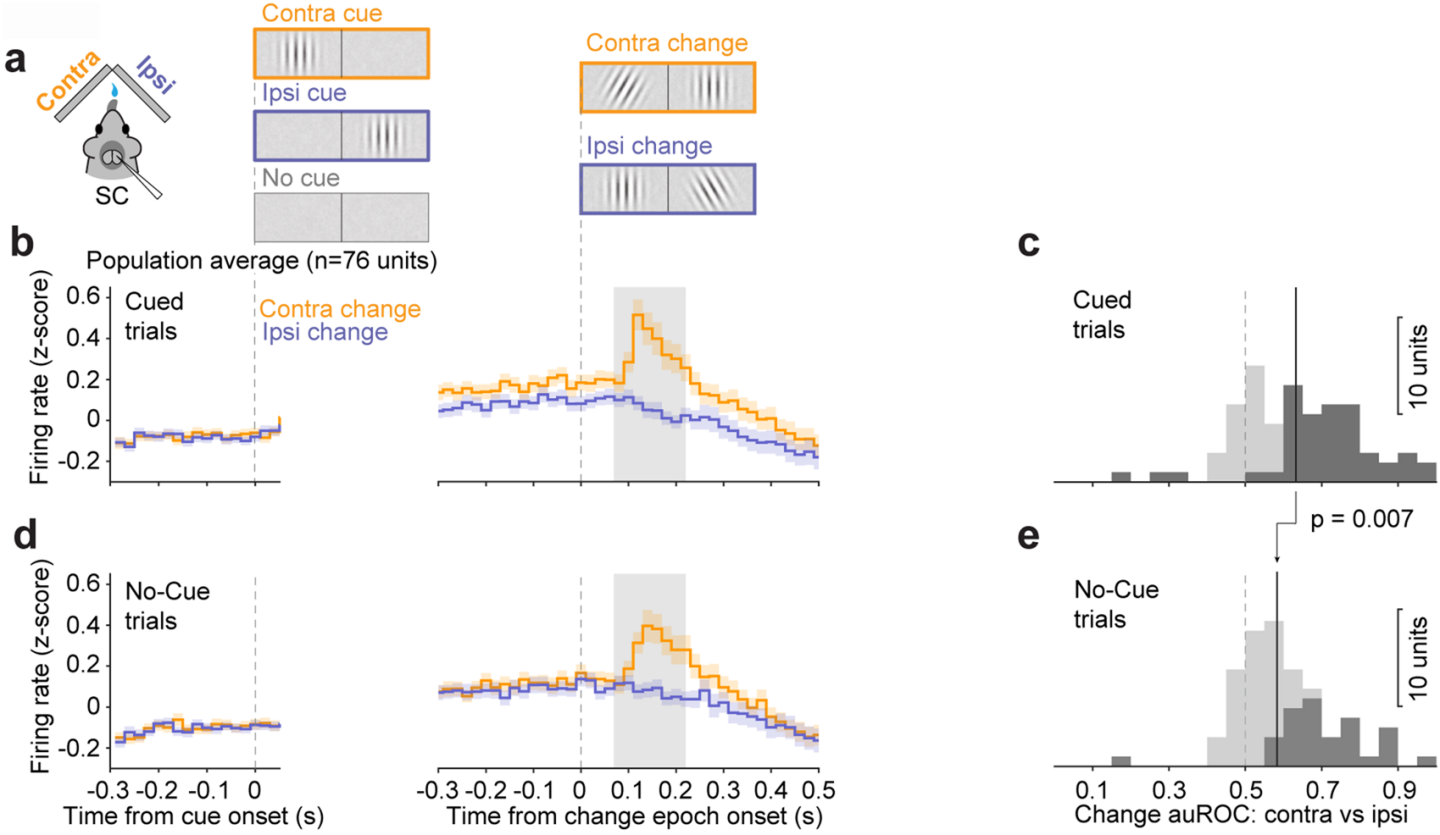
Spatial cueing can improve the detection of orientation changes if neuronal activity is compared across the contralateral and ipsilateral SC. (**a**) Schematic of apparatus and events in “cue/no-cue” task. (**b**) Average normalized population PSTHs of SC neurons during “cue-contra” change trials (orange) and “cue-ipsi” change trials (blue). Traces are aligned on the onset of the cue epoch (left panel, highlighting pre-cue baseline activity) and onset of the orientation change (right panel). Gray area: 150 ms change interval (60–210 ms after change onset) used to compute auROC comparing contralateral and ipsilateral change trials. (**c**) Distribution of change auROCs in cued trials. Dark bars are units with auROCs significantly different from chance level. Dashed line indicates chance value of 0.5, solid line indicates population median (0.63). (**d**) Presentation as in (**b**) but comparing “no-cue contra” (orange) to “no-cue ipsi” (blue) change trials. (**e**) Presentation as in (**c**), but in no-cue trials; (median = 0.58). The p value indicates comparison of population median between (**c**) and (**e**), paired-sample Wilcoxon signed-rank test.

Another possibility is that spatial cueing improves the ability of SC neuronal populations to discriminate change events in the relative responses of neurons in the contralateral versus ipsilateral halves of SC (Fig. 5a). To test this, we used unilateral recordings of SC activity obtained separately during contralateral and ipsilateral orientation changes as a proxy for simultaneous bilateral recordings during contralateral change events (Fig. 5b,d). Note that there was no difference in activity between the two colliculi before the onset of the visual stimuli (Fig. 5b,d left panels). We again computed auROC values for the “change” window (Fig. 5b,d, right panels), this time comparing contra-change to ipsi-change spike counts in with-cue trials, and separately for counts in no-cue trials. This analysis revealed that auROC values were significantly higher in with-cue trials compared to no-cue trials (p = 0.007, Fig. 5c,e). In addition, a larger proportion of SC neurons displayed significant auROC values (bootstrapped 95% CI ⊄ 0.5) in cued trials (31/76) than in no-cue trials (19/76; χ-square test; p = 0.038). Therefore, spatial cueing significantly improved SC neuronal discriminability when the relative levels of activity in the two colliculi were taken into consideration.

Although the primary effect of spatial cueing in our task was elevation of SC activity at the cued location during the delay and change epochs, we found evidence for enhanced suppression with cueing specifically during the orientation change. Comparison of spike counts during the change showed higher activity for contralateral changes on cue-contra trials compared to no-cue trials, as described above, and also slightly but significantly lower activity for ipsilateral changes on cue-ipsi trials compared to no-cue trials (Figure S5). These findings suggest an interaction between cue-related and stimulus-change-related influences on SC neuronal activity.

Finally, the timing of peak neuronal modulation between the two colliculi was related to variations in behavioral reaction time. When individual trials were separated based on the lick reaction times, we found that the time of peak neuronal modulation occurred earlier on trials with faster lick reaction times, and that there was a significant positive correlation between the timing of neuronal modulation and behavioral reaction times (Figure S6).

Together, these results demonstrate that cue-related modulation in the mouse does not necessarily increase the ability of SC neurons to locally discriminate between change and no-change events in our task. Instead, spatial cueing primarily enhanced activity at the cued location so that neuronal discriminability was improved when comparing activity across both sides of the SC, and the timing of this discrimination was correlated with behavioral reaction times.

## Discussion

We found that neurons in the intermediate layers of the mouse SC display cue-related modulation during a covert visual selective attention task—notably, the perceptual benefits from spatial cueing were confirmed by improvements in detection of a near-threshold visual change. Cue-related modulation emerged as the time of the relevant visual event approached, consistent with the time course of attention allocation in other species^29,30^. The main feature of cue-related modulation was an increase in the spike rates of SC neurons when the contralateral visual field was cued, consistent with the retinotopic representation in SC^31^. In addition to effects on spike rate, spike count correlations between pairs of SC neurons were also lower with contralateral spatial cues. Furthermore, by comparing activity across attentional conditions, we determined that cue-related modulation in our task was due to enhancement of SC activity at the cued location rather than suppression of SC activity at the uncued location.

Our results demonstrate how neurons in the mouse SC can contribute to the mechanisms of visual selective attention—their spiking activity is selectively elevated with spatial cueing so that processing of visual information is biased in favor of spatial locations expected to contain relevant information. This biasing of SC activity does not alter the local neuronal discriminability of visual events, and hence would not increase the sensitivity of behavioral performance if activity from only one side or region of the SC were used to guide detection performance. Instead, this biasing enhances the relative activity between the cued location and locations represented elsewhere in the SC, thereby improving discriminability of the relevant event if the readout mechanism involved a bilateral or global comparison of activity across the entire SC, as has been observed in primate SC^32^. Thus, depending on the specifics of the readout mechanism and how signals from the SC are pooled and compared, the changes we observed with spatial cueing could be consistent with effects on either bias or sensitivity of the observer.

Stimulus competition is an important aspect of visual selective attention and the SC plays a crucial role in selecting target stimuli amongst competing distractors in many species^16,20,33^, presumably reflecting an evolutionarily conserved midbrain function^34,35^. In the primate, SC activity is modulated in a variety of paradigms that involve stimulus selection, including the selection of targets for orienting movements^36,37^ and also selection of visual stimuli in the absence of orienting movements^6,38^. Further evidence has been provided by causal manipulations of SC activity that result in major changes to which alternative the subject selects^12,39,40^. Our finding of enhanced spiking activity in favor of the cued stimulus suggests that similar competitive mechanisms also apply to the mouse SC.

However, the particular competitive mechanism that would explain our results is distinctive. There is compelling evidence that inhibitory feedback circuits to the SC (or optic tectum) play a key role in implementing stimulus selection, and these involve suppression of competing locations across the SC map^16,41^. The results we obtained with our visual attention task point to a different mechanism. By interleaving with-cue and no-cue trials, we determined that our cue-related modulation was almost entirely due to enhancement of visual activity at the cued location, rather than suppression at the uncued distractor location. During the delay period of our task, the steady-state epoch most often used to document attention-related modulation, we found enhanced activity at the cued location with no detectable suppression at the non-cued location. This enhancement of activity at the cued location continued through the behaviorally relevant visual change event, when we also found slightly enhanced suppression for ipsilateral change events. This enhancement of activity at the cued location during our task—with smaller or no changes at the uncued location—is not easily explained by broad inhibitory feedback but would be consistent with a more focused excitatory or disinhibitory circuit mechanism recruited by the spatial cue. For example, this might involve a disinhibitory mechanism from the substantia nigra to the SC, as we recently demonstrated that activity through the direct pathway of the basal ganglia is linked to allocation of spatial attention^42^.

Although the cue-related modulation in our task was primarily due to enhancement at the cued location, other aspects of our data are consistent with inhibitory mechanisms. Most notably, the onset of the ipsilateral visual patch produced a brief reduction of SC activity that transiently obscured the contralateral cue-related effect (e.g., Fig. 1). These transient stimulus-driven suppressive effects could be mediated by inter-collicular inhibitory mechanisms^43^ or by inhibitory feedback projects from other brain regions^16,41^. With other task designs, especially those that would require actively ignoring competing stimuli^12,13^, these inhibitory mechanisms might also mediate larger cue-related suppression than what we observed and involve other circuit elements. For example, a recent study used auditory cues to direct visual attention in a “filter task” that required the mice to ignore the visual stimulus at the uncued location, and found that auditory inputs from the inferior colliculus during the delay period were crucial for task performance and attention-related modulation in the SC ^44^. Together, these findings illustrate how attention is an amalgam of multiple brain mechanisms, and we believe that comparisons across different types of attention tasks is a fruitful – and perhaps necessary – approach for functionally dissecting the complex, multi-component circuits involved.

The logic of the attention-related mechanism indicated by our results is also different from that described in visual cortex, because it involves a global comparison rather than a local improvement in discriminability. In visual cortex, it is thought that a local improvement of neuronal discriminability through sharpened visual tuning^45^, changes in receptive field properties^46^, and alterations in the statistical structure of activity amongst the population of active neurons^5^ all contribute to perceptual improvements at cued locations during the allocation of attention^28,47^. The lack of cue-related improvements in local neuronal discriminability of mouse SC neurons in our results might be related to how their activity is used in the task. Neurons in the SC are generally not selective to visual features or exhibit much broader tuning than visual cortical neurons^48^. Thus, the tuning of local pools of SC neurons for visual features might be less relevant for determining the accuracy of perceptual decisions; instead, the relative magnitude of activity across the SC retinotopic map might be much more important for setting the limits of detection performance^49^. This interpretation is consistent with previous studies showing that relative activity across the SC (or optic tectum) plays a central role in visual selection in fish^50^, birds^51^, and primates^24,32^. This type of mechanism is also reminiscent of computational models that use differential weighting of sensory evidence to explain how spatial cueing can account for the perceptual improvements during selective attention^52^. Thus, the relative activity across the SC might be a central component of the mechanism that implements visual spatial attention.

The effect of spatial cueing on interneuronal “noise” correlations we observed in mouse SC is consistent with previous observations in primate sensory cortical areas, but likely carries different functional implications. Pairs of neurons in primate visual cortex often display smaller correlations for cued stimuli than for uncued stimuli^47,53^, consistent with specific hypotheses about how the correlation structure of neuronal activity in these visual cortical areas impacts the decoding mechanism used to guide behavior^54,55^. Outside of cortical sensory areas, the possible importance of the neuronal correlation structure is less well established but also necessarily depends on how the neuronal activity is decoded^56,57^. Our hypothesis, that the detection of behaviorally relevant events involves global decoding of neurons across both the ipsilateral and contralateral SC rather than just activity at the contralateral site recruited by the stimulus event, implies a specific relationship between cue-related modulation of correlations for neuron pairs within and between the two halves of SC^32^. These predictions might be tested in future experiments that use simultaneous bilateral recordings in the mouse SC during visual attention tasks.

The control of attention by SC may interact with cortical mechanisms of attention. In mice, outputs from SC could significantly modulate visual cortical activity via the visual thalamus^58–61^. Notably, a recent study found spatially specific enhancement of activity in the mouse primary visual cortex during a block-organized visual detection task with target stimuli that occurred at one of two locations within the same hemifield^14^. However, it remains unclear how mouse visual cortical activity would be modulated in attention tasks like ours that include competing visual stimuli, which is a key factor for attention-related modulation in the primate visual cortex^62,63^.

The SC can also contribute to attention through circuits that operate downstream of cortical mechanisms. The basal ganglia were proposed as a possible candidate, based on their role in learned associations between visual events and behavioral responses^64^, and this has been supported by more recent findings. For instance, inactivation of the primate SC disrupts attention-related modulation of neuronal activity in caudate nucleus of the striatum^9,65^. In mice, it has been demonstrated that the dorsomedial striatum, putative homolog of the primate caudate nucleus, is causally involved in visual perceptual choices and visual selective attention^21,42^. Additional studies of the interactions between the SC, basal ganglia and cortical circuits will be necessary to understand how selective attention is implemented through the cooperative activity across these diverse brain regions.

## Methods

### Animals

All procedures were conducted on wild-type C57BL/6 J mice (JAX stock # 000664, Jackson Laboratory, Bar Harbor, ME, USA). The mice were housed in a 12:12 reversed day-night cycle, with lights off at 9 am, and all experimental procedures and behavioral training were done in the lights-off portion of the cycle (9am-9 pm). Two male and two female mice weighing 18–25 g were surgically implanted at age 6–8 weeks and then used in experiments for up to ~ 9 months. All the mice were in group housing (2–4 cage mates) prior to the surgical procedure, and subsequently singly housed after the implant surgery. All experimental procedures and animal husbandry were approved by the National Eye Institute Animal Care and Use Committee (ACUC) and complied with Public Health Service policy on the humane care and use of laboratory animals. The study was carried out in compliance with ARRIVE Guidelines.

All animals were used in the study, we did not exclude any mouse in the data collection. There was no animal group allocation in our sample as the experimental unit is neurons recorded from each animal. The number experimental units obtained from each animal and variability of measured properties were listed in the following section of Experimental Design and Data Analysis. Blinding was not relevant to our study as there was no group allocation. However, we randomized trial conditions within each block, as well as sequences for blocks across recording sessions to control for any potential biases from either the animal or investigator.

### Stereotaxic surgery

Each mouse was implanted with a head-holder before behavioral training; the procedure was similar to that in our previous study^13^. During the surgery, animals were anesthetized with isoflurane (4% induction, 0.8–1.5% maintenance) and secured by a stereotaxic frame with ear bars (Kopf Instruments, CA, USA). Dexamethasone (1.6 mg/kg) was administered to reduce inflammation. A feedback-controlled heating pad (PhysioSuite, Kent Scientific, CT, USA) was used to maintain the body temperature at 37 °C, and artificial tears were applied to the eyes to prevent them from drying. After the animal’s head was leveled in the stereotaxic frame, a scalp incision was made along the midline. A custom-designed titanium head post for head-fixing was positioned and secured to the skull using Metabond (Parkell Inc., NY, USA). The skin wound edge was then closed with sutures or tissue adhesive (Vetbond, 3 M, MN, USA). After surgery, mice received subcutaneous ketoprofen (1.85 mg/kg) daily for up to three days to alleviate any potential discomfort.

After each mouse was trained on the detection task for 20–30 days, a second surgery for implanting microwire bundles was carried out. Anesthesia procedure was identical to the first head-post surgery. After the animal’s head was leveled in the stereotaxic frame, a small craniotomy was made for implanting with moveable custom 16-wire microwire bundles (Innovative Neurophysiology, NC, USA). The coordinates for the tips of stainless steel cannula of the microwire bundles were ± 0.8 ~ 1.1 mm from midline (M-L axis), − 3.65 ~ − 4.0 mm from Bregma (A-P axis) and 0.2–0.5 mm ventral (D-V axis), based on a standard mouse brain atlas^66^. The cranial opening was sealed with bone wax and the microwire bundle assembly was secured on the skull with Metabond. Mice again received post-surgery subcutaneous ketoprofen (1.85 mg/kg) as needed to alleviate potential discomfort.

### Food control

After mice recovered from surgery and returned to above 95% of their pre-surgery weight (typically within 7–9 days), they were placed on a food control schedule. Mice had free access to water, but their intake of dry food was controlled, and they were allowed to augment their dietary intake by access to a nutritionally complete 8% soy-based infant formula (Similac, Abbott, IL, USA). Overall food intake was regulated to maintain at least 85% of their free-feeding body weight, and the health status of each mouse was monitored daily throughout the study. Mice were initially acclimatized to handling procedures by having their heads gently restrained while receiving the soy-based fluid under manual control via a sipper tube. After the initial exposure to soy-based fluid, the animal was more securely head-fixed, and manual delivery was continued. Once mice were adapted to these procedures, we switched to automatic delivery of fluid under computer control in the behavioral apparatus.

### Behavioral apparatus

The behavioral apparatus consisted of a custom-built booth that displayed visual stimuli to the mouse, the updating of the display was coupled to their locomotion. Details of apparatus construction are described elsewhere ^67^. The mouse was head-fixed in the center of the apparatus, positioned atop a polystyrene foam wheel (20-cm diameter) that allowed natural walking or running movements along a linear path. An optical encoder (Kübler, Germany) was used to measure the rotation of the wheel. The front walls of the booth incorporated a pair of LCD displays (VG2439, ViewSonic, CA, USA) positioned at 45° angles from the animal’s midline such that each display was centered on either the right or left eye and subtended ~ 90° horizontal by ~ 55° vertical of the visual hemifield, at a viewing distance of 27.5 cm. The interior of the booth was lined with sound absorbing material to reduce acoustic noise. The entire apparatus rested on a vibration isolation air table (Newport, CA, USA). The experiments were controlled by a computer using a modified version of the PLDAPS system^68^. Our system omitted the Plexon device, but included a Datapixx peripheral (Vpixx Technologies, Canada) and the Psychophysics Toolbox extensions^69,70^ for Matlab (The Mathworks, MA, USA), controlled by Matlab-based routines run on a Mac Pro (Apple Inc, CA, USA). The Datapixx device provided autonomous timing control of analog and digital inputs and outputs and synchronized the display of visual stimuli. A reward delivery spout was positioned near the snout of the mouse; lick contacts with the spout were detected by a piezo sensor (Mide Technology Co., MA, USA) and custom electronics. Each reward was a small volume (5–10 μl) of an 8% solution of soy-based infant formula (Similac, Abbott, IL, USA) delivered by a peristaltic pump (Harvard Apparatus, MA, USA) under computer and Datapixx control. The temperature inside the apparatus was maintained in a temperature range of 70–80° F.

### Visual detection tasks with spatial cueing

The tasks were similar to those we used previously^13,67^. Experiments were organized in blocks of randomly shuffled, interleaved trials, and each trial consisted of a sequence of epochs that the mouse progressed through by walking or running forwards on the wheel. Each epoch was defined by the particular stimuli presented on the visual displays, and the duration of each epoch was determined by the time required for the mouse to travel a randomized distance on the wheel. A typical trial lasted several seconds.

Each trial followed a standard sequence of four epochs. The average luminance across each visual display in all epochs was 4–8 cd/m^2^. In the first epoch (“noise”, not shown), the uniform gray of the inter-trial interval was replaced by pink noise with an RMS contrast of 3.3%; this epoch was presented for a wheel distance of 10–20 cm (range of time: 0.2–0.3 s). In the second epoch (“cue”), on cued trials a vertically oriented Gabor patch was added to the pink noise, centered in either the left or right visual display. The Gabor patch consisted of a sinusoidal grating (95% Michelson contrast) with a spatial frequency of 0.1 cycles per degree, a value chosen based on the visual spatial acuity of mice, modulated by a Gaussian envelope with full width at half-maximum of 18° ($\sigma$ = 7.5°). The phase of the grating was not fixed, but throughout the trial was incremented in proportion to the wheel rotation with every monitor refresh, so that the sinusoidal pattern was translated within the patch by approximately the same distance that the mouse traveled on the wheel; the Gabor patch on the left (right) drifted leftward (rightward), consistent with optic flow during locomotion. This second epoch lasted for 46–92 cm (0.36–1.55 s). On no-cue trials, no Gabor was added during the second epoch, but the otherwise the timing was the same. In the third epoch (“2 patch”), a second Gabor patch with the same spatial frequency and orientation appeared on the other side of the visual display; this epoch lasted for 107—214 cm (0.84—3.6 s). On no-cue trials, both Gabor patches appeared simultaneously in 2-patch epoch. The visual stimuli in the fourth epoch (“change”) depended on whether or not the trial included an orientation change. If the trial did include an orientation change, the cued Gabor patch changed orientation at the onset of the visual-event epoch; in no-cue trials, either one of the two Gabor patches changed its orientation with equal frequency. The amplitude of the orientation change was always 9°, which was near the detection threshold of mice. If the trial did not contain an orientation change, the two Gabor patches did not change their orientation, so that the “change” epoch unfolded as a seamless extension of the previous 2-patch epoch. Thus, in every experiment, the cue was always 100% valid, but an equal number of change and no-change trials were interleaved, making the probability of a change on any given trial 50% from the perspective of the subject.

The task of the mouse was to lick the spout when he or she detected a change in the orientation of the Gabor patch and to otherwise withhold from licking. Mice were required to lick within a 500-ms response window starting 300 ms after the orientation change in order to score a “hit” and receive a fluid reward. Any lick before the response window would result in trial abort and timeout penalty. If the mouse failed to lick within the response window after an orientation change, the trial was scored as a “miss” and no reward was given but no other penalty was applied. On “no change” trials, if the mouse licked within the same response window aligned on the unmarked transition to the fourth epoch, the trial was scored as a “false alarm”, which led to timeouts; if they correctly withheld from licking throughout the entire “change” epoch, the trial was scored as a “correct reject”. At the end of correct reject trials, the trial was extended to include an additional “safety-net epoch” in which the cued Gabor patch underwent a supra-threshold (30°) orientation change and the mouse could receive a reward by licking within a comparable response window. Responses in the safety-net epoch were not used for any analysis in the study.

Both variants of spatial cueing task experiments were organized as blocks of trials, with the sub-block conditions defined based on our recording site in the SC. In the “contra/ipsi cue” task variant, each block contained 80 trials, subdivided into 40 contralateral-cue trials (i.e., contralateral to our recording site in the SC), 40 ipsilateral-cue trials. The 40 contralateral-cue and 40 ipsilateral-cue trials were run back-to-back, with the order of the two sub-blocks randomly determined at the beginning of each single session. In the “cue/no-cue” task variant, each block contained 160 trials, subdivided into 40 contralateral-cue trials, 40 ipsilateral-cue trials, and 80 no-cue trials. The 40 contralateral-cue and 40 ipsilateral-cue trials were also run back-to-back, with the order of the two sub-blocks randomly determined in a given session. The sub-block of 80 no-cue trials were run either before or after the 80 (i.e., 40 plus 40) cue trials with equal probability. Of all trial types, 50% were with orientation change, randomly interleaved with no change trials. During a daily session, mice typically completed 320—800 trials in total.

### Electrophysiological recording

Spiking activity of SC neurons was recorded in four C57BL/6 J mice (2 males, 2 females) implanted with moveable 16-wire microwire bundles (Innovative Neurophysiology, NC, USA). Each wire comprised a 23 μm tungsten electrode. Electrophysiological signals were acquired through an RZ5D processor and Synapse Suite interface (Tucker-Davis Technologies, FL, USA) with voltages band-pass filtered (0.3 to 7 kHz) and sampled at 25 k Hz. The bundles were lowered along the dorsal–ventral axis with a microdrive included as part of the bundle assembly. The wire bundle was advanced 100–150 μm across successive recording sessions to isolate activity from additional units at different depths. Single units were sorted offline using KiloSort^71^.

In this study, we focused on neurons recorded in the intermediate and deep layers of the SC, defined as units identified at least 400 μm below the SC surface and no deeper than 2 mm. The SC surface was identified as the depth at which multi-unit visual responses across channels were first encountered. We reconstructed the bundle track in the SC of each mouse by identifying the gliosis in postmortem Nissl-stained tissue. Each relevant brain section was aligned on the standard mouse brain atlas^66^, and the SC depth of each recorded unit was then estimated based on the calibrated distance along the bundle track.

Firing rates of individual neurons were represented as peristimulus time histograms (PSTHs), using 20 ms non-overlapping bins aligned to the onset of task epochs. Normalization of spike rates for each neuron was done by subtracting the mean spike count from the PSTH and dividing by the standard deviation, using the mean and standard deviation of spike counts calculated from 20 ms bins across the entire recording session. The calculation of interneuronal spike-count correlations followed previously described procedures ^25^. Briefly, correlations were measured from simultaneously isolated pairs of single units with mean spike rates of at least 5 spikes/second. The Pearson correlation was computed for spike counts across trials measured during the final 200 ms of the 2-patch epoch (“delay period”).

### Mapping of visual receptive fields

SC units (n = 415 units) were further sub-selected based on having visual receptive fields that overlapped with the Gabor patch. Each visual attention task session was followed by a receptive field mapping session, during which white circular disks (118.5 cd/m^2^) of 10° in diameter were flashed against a gray background (7.2 cd/m^2^) in the visual display contralateral to the recording side. We sampled visual locations pseudo-randomly drawn from a 3 × 7 isotropic grid that extended from − 25° to 25° in elevation and 0° to 90° in azimuth of the contralateral visual field. An individual trial consisted of 8 consecutive 250 ms flashes, with each flash followed by a 250 ms blank period. At least 15 flash repetitions at each grid location were presented in each mapping session.

Receptive fields of individual neurons were estimated from the mean spike counts 50 to 150 ms after flash onset in each grid location, after subtracting baseline activity. The baseline in each trial was defined as the mean spike count within the 100-ms period before the presentation of the first flash. Baseline-subtracted mean spike counts were normalized by dividing by the maximum value evoked across grid locations. Normalized counts were linearly interpolated between grid locations with 1° resolution using the *scatteredInterpolant* function in Matlab and smoothed with a 2D Gaussian kernel ($\sigma$_x_ = $\sigma$_y_ = 5°); we defined the receptive field boundary as the isocline at 50% of the maximum value of the smoothed, interpolated normalized counts. The area of intersection (*S*_*i*_) between the receptive field (*S*_*r*_) and the Gabor patch (*S*_*g*_) was used to calculate an overlap ratio (*R*), defined as *R* = ((*S*_*i*_ / *S*_*r*_)^2^ + (*S*_*i*_ / *S*_*g*_)^2^)^1/2^. Only units (n = 311 units) with *R* > 0.25 were used for further analysis.

### Histology

Mice were euthanized with CO_2_ (1.0 LPM), after which they were transcardially perfused with ice-cold saline followed by phosphate buffered saline with 4% paraformaldehyde (PFA). Their brains were removed and stored in the 4% PFA solution overnight, and then transferred to a phosphate buffered saline solution for at least two days prior to sectioning. 80-µm sections were cut coronally using a vibratome (Campden Instruments, Ltd. England), and free-floating sections were processed for Nissl staining mounted on gelatin-coated glass slide. To visualize the tissue lesion caused by microwire bundle entries, sections were imaged under bright field at 10 × using a Zeiss fluorescent microscope to produce a whole brain reconstruction from tiled images of coronal sections.

Individual tiled images of coronal sections were aligned on the coordinates of the standard mouse brain atlas^66^, lesion tracks on each section were manually identified. Identified lesion tracks of each animal were all contained within 300 μm along either Anterior–Posterior (A-P) or Medial–Lateral (M-L) axis. A straight line perpendicular to the horizontal plane through the A-P and M-L coordinates which were used for the microwire bundle implantation was drawn. The straight line was used as the averaged bundle track only when it went through the lesion area. In all mice used in the study, the lines went through the identified lesion area. The putative locations of recorded neurons used in the study were projected onto the coronal plane of the standard mouse brain atlas with the nearest A-P coordinate that the averaged track went through.

### Monitoring mouse eye movements and pupil size

A high speed, 240 Hz CCD camera (ISCAN, MA, USA) was used to monitor eye position and pupil size of head-fixed mice during the entirety of the electrophysiology experiments. We imaged an area of 1.5 mm × 3 mm with a macro lens (ISCAN, MA, USA) centered on the eye. Four infrared light-emitting-diodes (wavelength 940 nm) were used to illuminate the eye. Commercially available acquisition software (ETL-200, ISCAN) was used to determine the center and boundary of the pupil. Gaze position was obtained by subtracting the center of corneal reflection from the pupil center to compensate any translational movement of the eye in the imaging plane. The gaze displacement rectilinear coordinates was converted to a rotation angle based on estimated eyeball radius (1.25 mm) from model C57bl/6 mice ^72^.

### Experimental design and statistical analysis

Data were obtained from a total of four C57BL/6 J mice in the study, two were male and two were females. We did not observe any systematic difference in behavioral performance between sexes in this study.

To verify the behavioral cueing effect, we tabulated hit and false alarm rates based on the definitions of trial outcomes described for the behavioral tasks, separately for each behavioral session. Performance was then characterized by measuring sensitivity (d’) and criterion using methods from signal detection theory^73^, as follows: d’ = Φ^-1^ (H) – Φ^-1^ (F), criterion = − (Φ^-1^ (H) + Φ^-1^ (F))/2, where Φ^-1^ is the inverse of the Gaussian cumulative distribution function, H is the hit rate and F is the false alarm rate. The 95% confidence intervals (CIs) of hit and false alarm rates were computed with the *binofit* function in Matlab, which uses the Clopper-Pearson method. The 95% CIs on d’ and criterion were computed with bootstrapped resampling. Unit spiking data from all completed trials regardless of behavioral outcome were included in our analysis.

The time course of each neuron’s cue-related modulation was computed from spike counts in non-overlapping 20 ms bins (aligned on specific epochs). For each unit, the area under the receiver operating characteristic curve (auROC) was calculated between spike counts in each bin for “cue-contra” trials and counts for “cue-ipsi” trials, following methods described previously^6^. For each 20-ms bin, two-tailed nonparametric Wilcoxon signed-rank tests were performed to determine whether the distributions of population auROC values in that particular bin had medians significantly different from 0.5 null value.

For the pre-cue baseline activity, spike counts from a 200-ms bin immediately preceding the cue epoch onset from each trial were used in two different ways. First, we computed auROC values for each neuron as described above. A bootstrapping procedure was used to compute the 95% CIs of the baseline auROC, and if the CI was completely above or below 0.5, the unit was considered significantly modulated. Second, we computed the mean spike count in this baseline period in the same cue condition for each neuron and then ran a population paired-sample Wilcoxon signed rank test between “contra cue” and “ipsi cue” conditions in “contra/ipsi cue” datasets, or non-parametric Kruskal–Wallis one-way ANOVA among cueing conditions in “cue/no-cue” datasets.

For the cue-related modulation during the “delay-period” of “contra/ipsi cue” experiments, spike counts from a 200-ms bin immediately preceding the change epoch from each trial were used in two different ways. First, we computed auROC values for each neuron as described above. A bootstrapping procedure was used to compute the 95% CIs of the delay-period auROC, and if the CI was completely above or below 0.5, the unit was considered significantly modulated. Second, we computed an attention modulation index (AMI) for each unit from mean spike counts in contra-cue trials (*Count*_*contra*_) and ipsi-cue trials (*Count*_*ipsi*_) in the 200 ms epoch: AMI = (*Count*_*contra*_ – *Count*_*ipsi*_) / (*Count*_*contra*_ + *Count*_*ipsi*_). Nonparametric rank sum tests were performed for each unit to compare spike counts in each 200 ms bin between contra-cue and ipsi-cue trials; a unit with p < 0.05 was considered to have a significant AMI. The number of units included from each animal and sex, and their mean delay period auROC values were as follows: #E02 (male, 112 units, auROC: 0.555 ± 0.064, mean ± STD); #E03 (female, 60 units, auROC: 0.576 ± 0.098); #E05 (female, 109 units, auROC: 0.519 ± 0.085); #E06 (male, 30 units, auROC: 0.501 ± 0.059).

For the cue-related modulation during the “delay-period” of “cue/no-cue” experiments, spike counts from the 200-ms delay period from each trial were used in three different ways. In addition to computing auROC and AMI values as described above, spike counts from the “delay period” of individual trials were used for two-way ANOVA analysis of variance, taking the trial-by-trial variability into account, which was also featured in the auROC analysis. The ANOVA analysis used “anovan.m” function in Matlab, with two grouping factors: 1) cueing conditions (contra-cue, ipsi-cue and no-cue) and 2) unit identity (ID of 76 units used in the analysis). Tukey–Kramer post-hoc comparisons after the ANOVA were used to compare the spike-count differences across cueing conditions.

For the visual change-related activity, we performed the same analyses on the spike counts in the interval 60–210 ms after the change in orientation of the Gabor patch, computing auROC values for each neuron by comparing spike counts across trial conditions indicated in the main text (Figs. 4,5). Confidence intervals and associated statistical significance were again determined using a bootstrapping procedure.

For the linear regression analysis of factors contributing to spike rate variability (Figs. 1,2), we used Matlab function “*fitlm.m”* to model the observed spike rates in each neuron using three predictors: cue location, running speed and pupil size. We discretized running speed and pupil size, making them categorical variables. Normalized spike rate (z-scored) in four 200 ms intervals were modeled. Cue: from cue onset; early 2-patch: from 2-patch onset; mid 2-patch: from 250 ms after; delay: final 200 ms of 2-patch. Coefficients from the model fits represent the weights from each predictor that best explained the spike rate variability. The p value for testing the null hypothesis whether a predictor’s coefficient was equal to zero came from *t-statistic* for the model fit of individual unit, the degrees of freedom depended on trial counts from each recording session, between 300 and 800. Post hoc multiple comparisons with Tukey–Kramer correction after one-way ANOVA (degrees of freedom for groups: 2; degrees of freedom for errors, 930; F = 27.07) were used to assess the differences of predictor coefficient values during the delay period.

To analyze the correlation between neuronal responses and behavioral reaction time (RT), we measured RTs and firing rates from hit trials (i.e., trials with an RT), separately for cued and no-cue trials. For each individual session, we split the RTs into “slow” and “fast” trials (based on the median RT) and computed separate means over “slow” and “fast” RTs. Next, we computed the mean z-scored firing rate over time (PSTH) separately based on whether the change event was contra or ipsi and whether the RT was fast or slow. We then took the difference in firing rate between contra change and ipsi change separately for trials with fast and slow RTs and identified the time at which these firing rate differences reached their maximum value for each neuron. These times were defined as the “latency of peak neuronal modulation” that we’ll also refer to as “neuronal RT” for short. For each unit, the Pearson’s correlation coefficient between the neuronal RTs and the mean behavioral RTs of the recording session was computed. In the results reported in Figure S6, each unit contributes to two data points: faster trials and slower trials.

Statistical analyses were conducted in Matlab using the statistics and machine learning toolbox, and statistical significance was defined as *p* < 0.05 unless otherwise noted. Nonparametric rank-sum tests were computed using spike counts from − 200 to 0 ms before cue epoch onset between contralateral and ipsilateral trials to determine whether a unit display significant spatial modulation before the presence of the spatial cue. We used spike counts from four different time windows in the detection task to determine whether a given unit had a significant response to the onset of visual epochs: base (− 100 ms to 0 ms from cue onset), cue (+ 50 ms to + 150 ms from cue onset), late-2 (− 100 ms to 0 ms from change onset), change (+ 50 ms to + 150 ms from change onset). Nonparametric rank-sum tests were computed comparing spike rates in base to cue windows to determine whether a unit had a significant response to the cue. Nonparametric rank-sum tests were computed comparing spike rates in the late-2 to change windows to determine whether a unit had significant responses to the visual change. One-tailed nonparametric Wilcoxon signed-rank tests were performed to determine whether the distributions of population auROC values had medians significantly larger than 0.5, and whether the distributions of population AMI had medians significantly larger than 0. Paired-sample nonparametric Wilcoxon signed-rank tests were performed to compare the effect of spatial cueing on behavioral d’ and criterion across sessions (n = 25). Paired-sample nonparametric Wilcoxon signed-rank tests were performed to compare the effect of spatial-cue locations on neuronal discriminability (change-related auROC) and spike count of visual change at the “change window”. χ-square tests were performed to compare proportions of units with significant change-related auROC values across different cueing conditions. Paired-sample nonparametric Wilcoxon signed-rank tests were performed to compare the effect of spatial-cue locations on interneuronal spike count correlations across the population. The value of n reported in the figures and results indicates the number of units or unit-pairs. Error bars in figures indicate 95% CI on the median or mean, unless indicated otherwise.

## Supplementary Information

Supplementary Information.

## Data Availability

All of the data were acquired and initially processed using custom scripts written in Matlab (The Mathworks, MA). The Matlab code and data that support the findings of this study will be made available from the corresponding author upon reasonable request.

## References

[1] Desimone R, Duncan J (1995). Neural mechanisms of selective visual attention. Annu. Rev. Neurosci..

[2] Squire RF, Noudoost B, Schafer RJ, Moore T (2013). Prefrontal contributions to visual selective attention. Annu. Rev. Neurosci..

[3] Bisley JW, Goldberg ME (2010). Attention, intention, and priority in the parietal lobe. Annu. Rev. Neurosci..

[4] Reynolds JH, Chelazzi L (2004). Attentional modulation of visual processing. Annu. Rev. Neurosci..

[5] Cohen MR, Maunsell JHR (2009). Attention improves performance primarily by reducing interneuronal correlations. Nat. Neurosci..

[6] Herman, J. P. & Krauzlis, R. J. Color-change detection activity in the primate superior colliculus. *eNeuro* **4,** ENEURO.0046–17.2017 (2017). 10.1523/ENEURO.0046-17.2017 PMC5388837 28413825

[7] Briggs F, Mangun GR, Usrey WM (2013). Attention enhances synaptic efficacy and the signal-to-noise ratio in neural circuits. Nature.

[8] McAlonan K, Cavanaugh J, Wurtz RH (2008). Guarding the gateway to cortex with attention in visual thalamus. Nature.

[9] Arcizet F, Krauzlis RJ (2018). Covert spatial selection in primate basal ganglia. PLoS Biol.

[10] Krauzlis RJ, Lovejoy LP, Zénon A (2013). Superior colliculus and visual spatial attention. Annu. Rev. Neurosci..

[11] Snow JC, Allen HA, Rafal RD, Humphreys GW (2009). Impaired attentional selection following lesions to human pulvinar: evidence for homology between human and monkey. Proc. Natl. Acad. Sci. USA.

[12] Lovejoy LP, Krauzlis RJ (2010). Inactivation of primate superior colliculus impairs covert selection of signals for perceptual judgments. Nat. Neurosci..

[13] Wang L, Krauzlis RJ (2018). Visual selective attention in mice. Curr. Biol..

[14] Speed A, Del Rosario J, Mikail N, Haider B (2020). Spatial attention enhances network, cellular and subthreshold responses in mouse visual cortex. Nat. Comms..

[15] You W-K, Mysore SP (2020). Endogenous and exogenous control of visuospatial selective attention in freely behaving mice. Nat. Comms..

[16] Mysore SP, Knudsen EI (2011). The role of a midbrain network in competitive stimulus selection. Curr. Opin. Neurobiol..

[17] Goldberg ME, Wurtz RH (1972). Activity of superior colliculus in behaving monkey. II. Effect of attention on neuronal responses. J. Neurophysiol..

[18] Ito S, Feldheim DA (2018). The mouse superior colliculus: An emerging model for studying circuit formation and function. Front. Neural Circuits.

[19] Wang L, McAlonan K, Goldstein S, Gerfen CR, Krauzlis RJ (2020). A Causal role for mouse superior colliculus in visual perceptual decision-making. J. Neurosci..

[20] Lee KH, Tran A, Turan Z, Meister M (2020). The sifting of visual information in the superior colliculus. Life.

[21] Wang L, Rangarajan KV, Gerfen CR, Krauzlis RJ (2018). Activation of striatal neurons causes a perceptual decision bias during visual change detection in mice. Neuron.

[22] Luck SJ, Chelazzi L, Hillyard SA, Desimone R (1997). Neural mechanisms of spatial selective attention in areas V1, V2, and V4 of macaque visual cortex. J. Neurophysiol..

[23] Green DM, Swets JA (1966). Signal detection theory and psychophysics.

[24] Kim B, Basso MA (2008). Saccade target selection in the superior colliculus: A signal detection theory approach. J. Neurosci..

[25] Mitchell JF, Sundberg KA, Reynolds JH (2009). Spatial attention decorrelates intrinsic activity fluctuations in macaque area V4. Neuron.

[26] Savier EL, Chen H, Cang J (2019). Effects of locomotion on visual responses in the mouse superior colliculus. J. Neurosci..

[27] Schröder S (2020). Arousal modulates retinal output. Neuron.

[28] Reynolds JH, Heeger DJ (2009). The normalization model of attention. Neuron.

[29] Egeth HE, Yantis S (1997). Visual attention: control, representation, and time course. Annu Rev Psychol.

[30] Knudsen EI (2007). Fundamental components of attention. Annu. Rev. Neurosci..

[31] Dräger UC, Hubel DH (1976). Topography of visual and somatosensory projections to mouse superior colliculus. J. Neurophysiol..

[32] Herman JP, Katz LN, Krauzlis RJ (2018). Midbrain activity can explain perceptual decisions during an attention task. Nat. Neurosci..

[33] Nummela SU, Krauzlis RJ (2010). Inactivation of primate superior colliculus biases target choice for smooth pursuit, saccades, and button press responses. J. Neurophysiol..

[34] Krauzlis RJ, Bogadhi AR, Herman JP, Bollimunta A (2018). Selective attention without a neocortex. Cortex.

[35] Knudsen EI (2020). Evolution of neural processing for visual perception in vertebrates. J. Comp. Neurol..

[36] McPeek RM, Keller E (2002). Saccade target selection in the superior colliculus during a visual search task. J. Neurophysiol..

[37] Krauzlis R, Dill N (2002). Neural correlates of target choice for pursuit and saccades in the primate superior colliculus. Neuron.

[38] Ignashchenkova A, Dicke PW, Haarmeier T, Thier P (2004). Neuron-specific contribution of the superior colliculus to overt and covert shifts of attention. Nat. Neurosci..

[39] McPeek RM, Keller EL (2004). Deficits in saccade target selection after inactivation of superior colliculus. Nat. Neurosci..

[40] Carello CD, Krauzlis RJ (2004). Manipulating intent: evidence for a causal role of the superior colliculus in target selection. Neuron.

[41] Mysore SP, Knudsen EI (2013). A shared inhibitory circuit for both exogenous and endogenous control of stimulus selection. Nat. Neurosci..

[42] Wang L, Krauzlis RJ (2020). Involvement of striatal direct pathway in visual spatial attention in mice. Curr. Biol..

[43] Essig J, Hunt JB, Felsen G (2021). Inhibitory neurons in the superior colliculus mediate selection of spatially-directed movements. Commun. Biol..

[44] Hu F, Dan Y (2021). An inferior-superior colliculus circuit controls auditory cue-directed visual spatial attention. Neuron.

[45] Martinez-Trujillo JC, Treue S (2004). Feature-based attention increases the selectivity of population responses in primate visual cortex. Curr. Biol..

[46] Womelsdorf T, Anton-Erxleben K, Pieper F, Treue S (2006). Dynamic shifts of visual receptive fields in cortical area MT by spatial attention. Nat. Neurosci..

[47] Maunsell JHR (2015). Neuronal mechanisms of visual attention. Annu. Rev. Vis. Sci..

[48] Wang L, Sarnaik R, Rangarajan K, Liu X, Cang J (2010). Visual receptive field properties of neurons in the superficial superior colliculus of the mouse. J. Neurosci..

[49] Boehnke SE, Munoz DP (2008). On the importance of the transient visual response in the superior colliculus. Curr. Opin. Neurobiol..

[50] Fernandes AM (2020). Neural circuitry for stimulus selection in the zebrafish visual system. Neuron.

[51] Mysore SP, Knudsen EI (2011). Flexible categorization of relative stimulus strength by the optic tectum. J. Neurosci..

[52] Eckstein MP, Peterson MF, Pham BT, Droll JA (2009). Statistical decision theory to relate neurons to behavior in the study of covert visual attention. Vision. Res..

[53] Nienborg H, Cohen MR, Cumming BG (2012). Decision-related activity in sensory neurons: Correlations among neurons and with behavior. Annu. Rev. Neurosci..

[54] Ruff DA, Ni AM, Cohen MR (2018). Cognition as a window into neuronal population space. Annu. Rev. Neurosci..

[55] Kohn A, Coen-Cagli R, Kanitscheider I, Pouget A (2016). Correlations and neuronal population information. Annu. Rev. Neurosci..

[56] Tremblay S, Pieper F, Sachs A, Martinez-Trujillo J (2015). Attentional filtering of visual information by neuronal ensembles in the primate lateral prefrontal cortex. Neuron.

[57] Bartolo R, Saunders RC, Mitz AR, Averbeck BB (2020). Information-limiting correlations in large neural populations. J. Neurosci..

[58] Beltramo R, Scanziani M (2019). A collicular visual cortex: Neocortical space for an ancient midbrain visual structure. Science.

[59] Ahmadlou M, Zweifel LS, Heimel JA (2018). Functional modulation of primary visual cortex by the superior colliculus in the mouse. Nat. Comms..

[60] Hu F (2019). Prefrontal Corticotectal Neurons enhance visual processing through the superior Colliculus and Pulvinar Thalamus. Neuron.

[61] Tohmi M, Meguro R, Tsukano H, Hishida R, Shibuki K (2014). The Extrageniculate visual pathway generates distinct response properties in the higher visual areas of mice. Curr. Biol..

[62] Desimone R (1998). Visual attention mediated by biased competition in extrastriate visual cortex. Philos. Trans. R. Soc. Lond. B Biol. Sci..

[63] Beck DM, Kastner S (2005). Stimulus context modulates competition in human extrastriate cortex. Nat. Neurosci..

[64] Krauzlis RJ, Bollimunta A, Arcizet F, Wang L (2014). Attention as an effect not a cause. Trends Cogn. Sci..

[65] Herman JP, Arcizet F, Krauzlis RJ (2020). Attention-related modulation of caudate neurons depends on superior colliculus activity. Elife.

[66] Paxinos G, Franklin K (2019). Paxinos and Franklin's the mouse brain in stereotaxic coordinates.

[67] Krauzlis RJ (2020). Visual Psychophysics in Head-Fixed Mice. Curr. Protoc. Neurosci..

[68] Eastman KM, Huk AC (2012). PLDAPS: a hardware architecture and software toolbox for neurophysiology requiring complex visual stimuli and online behavioral control. Front. Neuroinform..

[69] Brainard DH (1997). The psychophysics toolbox. Spat. Vis..

[70] Pelli DG (1997). The VideoToolbox software for visual psychophysics: transforming numbers into movies. Spat. Vis..

[71] Pachitariu, M., Steinmetz, N. A., Kadir, S., Carandini, M. & Harris, K. D. Kilosort: realtime spike-sorting for extracellular electrophysiology with hundreds of channels. *bioRixv* 1–9 (2016).

[72] Sakatani T, Isa T (2004). PC-based high-speed video-oculography for measuring rapid eye movements in mice. Neurosci. Res..

[73] Macmillan NA, Creelman CD (2005). Detection Theory: A User's Guide.

