# Supplementary material for: Neuronal modulation in the mouse superior colliculus during covert visual selective attention
Source: Sci Rep. 2022 Feb 15;12:2482. doi: 10.1038/s41598-022-06410-5 (PMC8847498; doi:10.1038/s41598-022-06410-5)
Supplement: Supplementary file 1 — Supplementary Information. [file 41598_2022_6410_MOESM1_ESM.pdf]

Neuronal modulation in the mouse superior colliculus during covert visual selective attention

Lupeng Wang[1*], James P. Herman[2], & Richard J. Krauzlis[1*]

1. Laboratory of Sensorimotor Research, National Eye Institute
Bethesda, Maryland 20892 USA

2. Department of Ophthalmology, University of Pittsburgh, Pittsburgh, PA 15213

Fig. S1

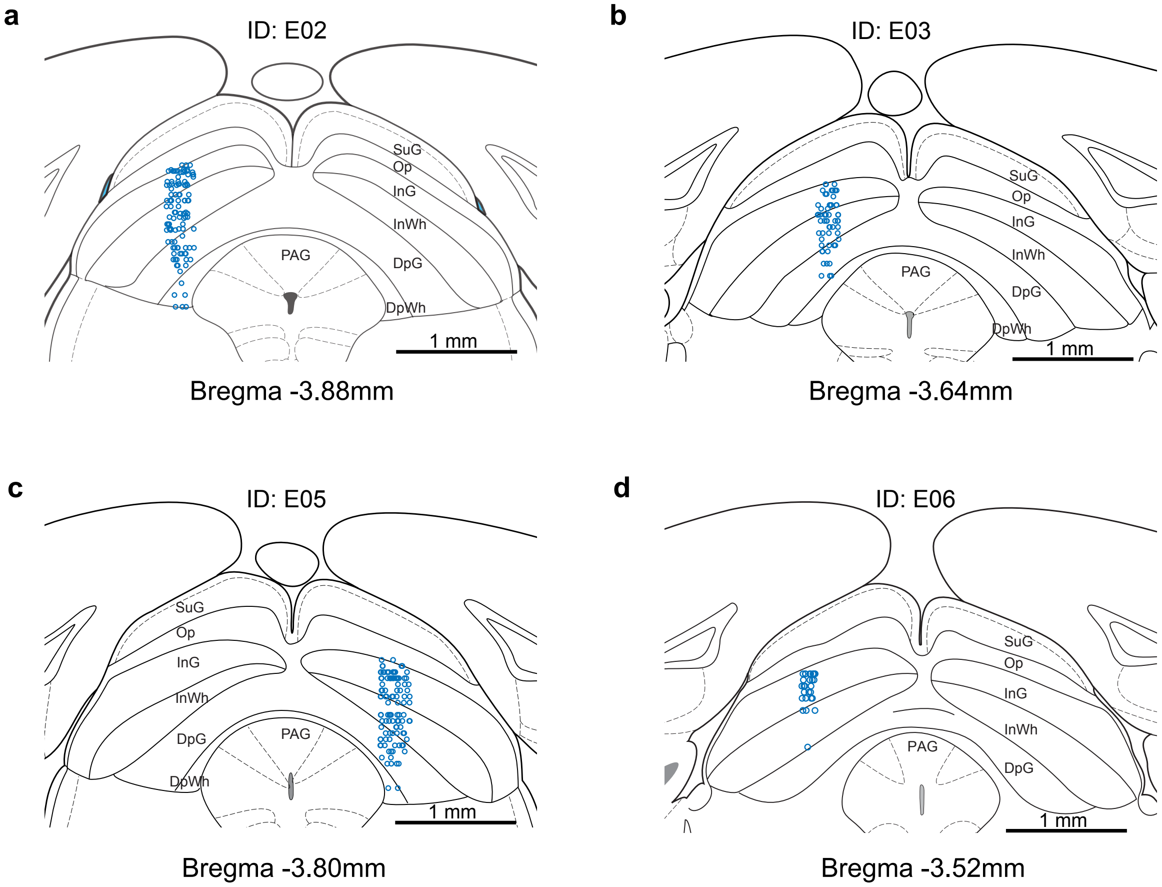

Fig. S1. **Summary of anatomical locations of recorded SC neurons**. **a**) Reconstructed locations of recorded SC neurons in a sample mouse (ID: E02) projected onto a standard Mouse Brain Atlas coronal plane at the anterior-posterior (A-P) coordinate of the reconstructed averaged bundle track. See Methods for details of the bundle track reconstruction. Only areas around the SC are shown in the coronal plane. Blue circles represent dorsal-ventral locations of individual neurons jittered at the medial-lateral (M-L) coordinate of the averaged bundle track. The disperse of the jitter is for illustration purpose and it is constrained with the extent of bundle track lesion revealed in postmortem histology. Scale bar: 1mm. SC layers abbreviations: SuG:

superficial grey layer; Op: optic layer; InG: intermediate grey layer; InWh: intermediate white layer; DpG: deep grey layer; DpWh: deep white layer. PAG: Peri Aquatic Grey. **b**) As in **a**, but for the SC neuronal location summary of the mouse E03. **c**) As in **a**, but for the SC neuronal location summary of the mouse E05. **d**) As in **a**, but for the SC neuronal location summary of the mouse E06.

Fig. S2

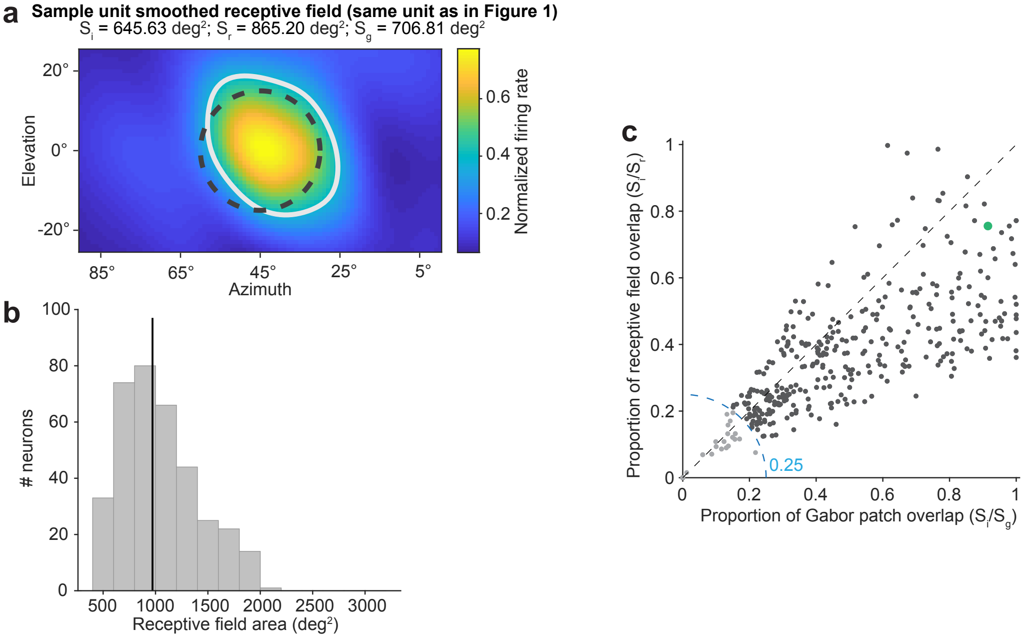

Fig. S2. Receptive fields of recorded SC neurons. a) Receptive field of sample neuron shown in Fig. 1**b**. Colormap: normalized activity evoked by flashed disks across spatial grids after smoothing with 2D Gaussian kernel (σ = 5°). Light gray contour line: area with at least 50% of peak activity in the smoothed map; black dashed circle: the location of Gabor patch. **b**) Distribution of receptive field size of mapped SC neurons, defined as area within the 50% contour line. Solid line: population median (971.4 deg^2). **c**) Scatter plot of receptive field and Gabor patch overlap ratio of individual neurons. X-axis: ratio of overlapped area divided by Gabor patch area; y-axis: ratio of overlapped area divided by receptive field area. Blue dash: radius of 0.25 overlap ratio (R) used as the inclusion criteria. Only units outside the radial arc (dark dots) were used for further analysis in the paper. The larger green dot is the same unit shown in panel **a**.

Fig. S3

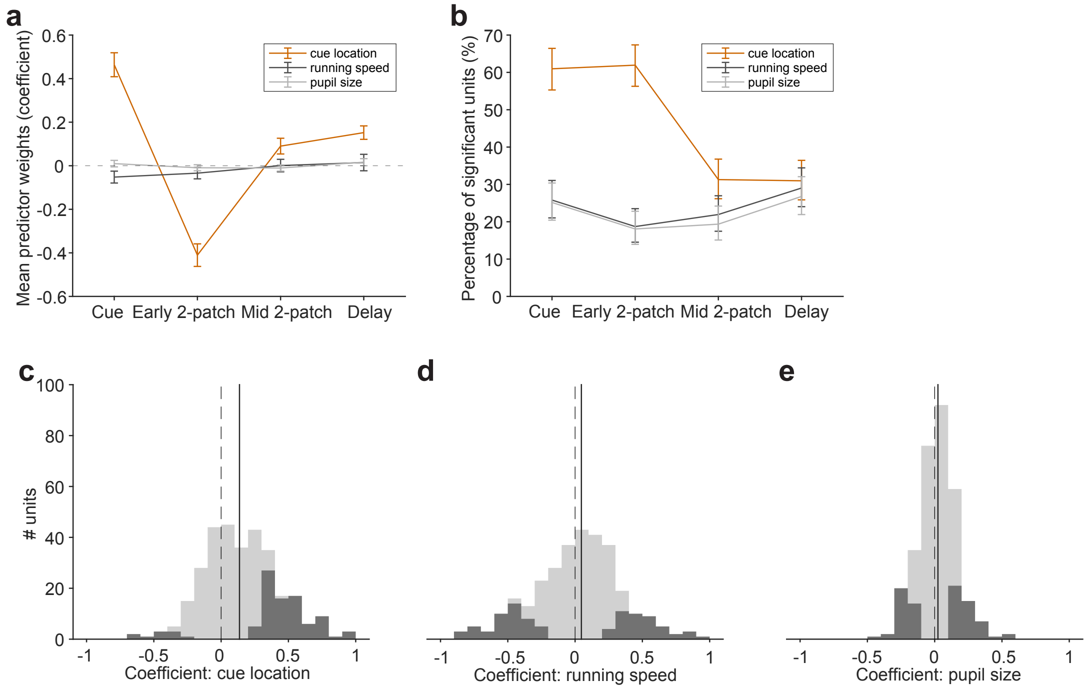

Fig. S3. Contribution of running speed, pupil size and cue location on SC activity during the task. a) Mean coefficient of linear regression of cue location (brown), running speed (black) and pupil size (gray) on variability of SC activity during different 200 ms epoch intervals. Cue: from cue onset; early 2-patch: from 2-patch onset; mid 2-patch: from 250ms after; delay: last 200ms of 2-patch. Errorbar: 95% CI. **b**) Similar to **a**, but for percentage of units significantly modulated by different predictors (p < 0.01, t-statistic to test the null hypothesis whether a predictor coefficient in the regression model is equal to zero for each unit, see Methods). **c**) Distribution of coefficient of cue location on SC activity during delay period. Dark bars are units with coefficient significantly different from zero. Dashed line indicates null value of 0, solid line indicates

population median (0.14). **d**) Presentation as in **c**, but for running speed (median = 0.05). **e**) Presentation as in **c**, but for pupil size (median = 0.02).

Fig. S4

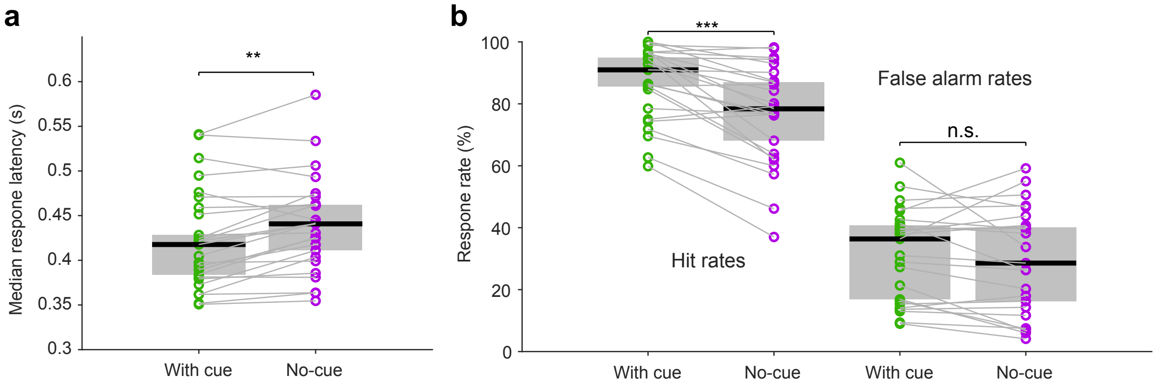

Fig. S4. Spatial cueing improves both reaction times and response accuracy of attention task performance. a) Reaction times of individual sessions for with-cue trials (green) and no-cue trials (purple). Lines connect the two types of trials within the same session, which was used for pairwise comparisons. Error bars show population median with 95% confidence intervals. **b**) Similar to **a**, but for response accuracy of individual sessions, showing both hit rates (left) and false alarm rates (right). ($^{***}p < 0.001$; $^{**}p < 0.01$; $^{n.s.}p = 0.15$, pairwise Wilcoxon signed-rank test)

Fig. S5

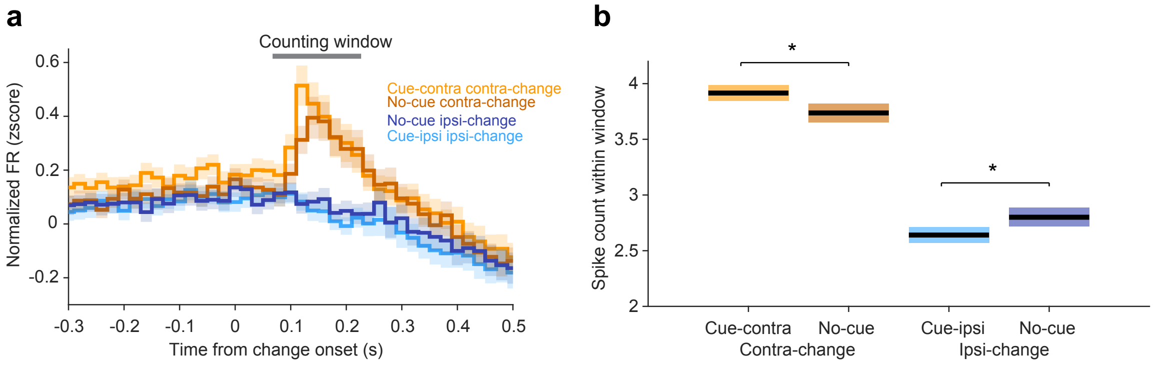

Fig. S5. Change-period spike count comparisons among all four trial types when an orientation change occurred. a) Overlay of normalized mean PSTHs of all four trial types during an orientation change to illustrate potential differences. All four PSTH traces and counting window are the same as in Figure 5. Error bars are 95% CI of the mean. **b**) Comparisons of spike counts within the 150-ms counting window (as in **a**) among all trial types. Error bars show mean spike count with 95% CI. (contra-change with cue-contra: 3.92 ± 0.07 spikes; contra-change no-cue: 3.74 ± 0.08 spikes; ipsi-change with cue-ipsi: 2.64 ± 0.07 spikes; ipsi-change no-cue: 2.80 ± 0.08 spikes; $^{*}p < 0.05$, Turkey-Kramer *post hoc* comparison following two-way ANOVA for trial-by-trial spike count comparisons, using trial-type conditions and neuron identity as factors)

Fig. S6

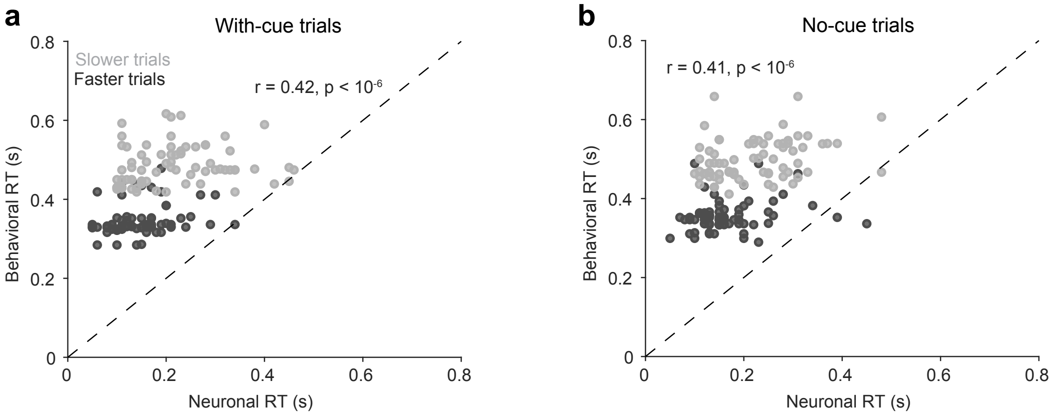

Fig. S6. Correlations between neuronal reaction times and behavioral reaction times of “cue/no-cue” dataset. a) Neuronal reaction times versus behavioral reaction times for with-cue trials. For each individual session, the RTs were binned into “slow” and “fast” (median split), and the mean for of “slow” (lighter) and “fast” (darker) RTs was separately computed. Next, separating the trials based on slow/fast RTs, the mean z-scored firing rate for contra change and ipsi change was computed. Then the difference between contra change and ipsi change was used to define for the “latency of peak neuronal modulation” that we’ll call neuronal RT for short. The neuronal RTs versus the mean behavioral RTs for all of our individual units are plotted. The neuronal RTs were faster in trials with faster behavioral RT (cued trials, neuronal RT mean: 0.146 s, SD: 0.061; behavioral RT, mean: 0.347 s, SD: 0.04) than in slower trials (cued trials, neuronal RT mean: 0.215 s, SD: 0.097; behavioral RT, mean: 0.458 s, SD: 0.05). It overall shows significant positive correlation when both faster and slower trials are included. **b**) Similar to **a**, but for no-cue trials.
